# Al─N Co‐Doped LLZO Solid Electrolytes via One‐Step Sintering: Toward High Ionic Conductivity

**DOI:** 10.1002/advs.75980

**Published:** 2026-06-09

**Authors:** Hao Zhang, Yaocong Wang, Quande Che, Jiaming Wu, Yanzhu Zhang, Dongxu Mao, Xundao Liu, Jiajie Li, Zhengmao Ye, Dehua Dong

**Affiliations:** ^1^ School of Materials Science and Engineering University of Jinan Jinan P. R. China; ^2^ School of Civil Engineering and Architecture Hainan University Haikou P. R. China; ^3^ School of Materials Science and Engineering Hainan University Haikou P. R. China; ^4^ Moganshan Institute ZJUT Deqing P. R. China

**Keywords:** Al─N co‐doped, ionic conductivity, Li^+^ migration, one‐step sintering

## Abstract

Garnet‐type Li_7_La_3_Zr_2_O_12_ (LLZO) is a promising solid electrolyte for solid‐state lithium batteries owing to its relatively high ionic conductivity and wide electrochemical window. However, its conductivity still needs further improvement to meet practical application requirements. Herein, we propose for the first time a cation–anion co‐doping strategy to synthesize Al─N co‐doped LLZO via a one‐step sintering process, achieving a high ionic conductivity of 2.19 × 10^−3^ S cm^−1^. Nudged elastic band (NEB) calculations reveal that Al─N co‐doping reduces the energy barrier for Li^+^ migration, thereby enhancing ionic transport. Remarkably, the Li|LLZO–Al_0.50_N_0.50_|Li symmetric cell demonstrates stable lithium plating/stripping cycling over 600 h at 0.1 mA cm^−2^, and the LiFePO_4_| LLZO–Al_0.50_N_0.50_|Li full cell retains 82.6% of its initial capacity after 200 cycles at 0.3 C. This work confirms Al─N co‐doping as an effective strategy for improving the ionic conductivity of LLZO, offering a viable route toward high‐performance garnet‐type solid electrolytes.

## Graphical abstract

Al–N co‐doped LLZO solid electrolytes were prepared via a one‐step sintering process, which not only simplifies fabrication process, but also diminishes Li loss during high‐temperature sintering in conventional fabrication process. The Al–N co‐doped LLZO achieves a high ionic conductivity of 2.19 × 10^−3^ S cm^−1^ because the co‐doping reduces the energy barrier for Li^+^ migration.

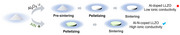

## 1 Introduction

Lithium‐ion batteries (LIBs) are extensively used in electric vehicles and portable electronics [1]. However, conventional LIBs employ flammable liquid electrolytes, leading to substantial safety risks [2], and thereby limiting their broader applications [3]. In contrast, solid‐state electrolytes (SSEs) present a promising alternative, offering the improved thermal stability, the higher energy density, and the wider operating temperature range [4, 5, 6]. As a result, SSEs are regarded as a crucial pathway toward developing next‐generation safe batteries [7, 8].

Li_7_La_3_Zr_2_O_12_ (LLZO) distinguishes itself among solid‐state electrolytes owing to the combination of high ionic conductivity, a wide electrochemical window, and excellent electrochemical stability [9, 10]. LLZO exhibits two distinct crystal phases: a tetragonal phase with an ordered lithium‐ion arrangement, and a cubic phase that is stable at high temperatures and features a disordered lithium distribution for fast ion conduction [9, 11]. Extensive research has been conducted to obtain the cubic phase with high ionic conductivities. For instance, the substitution of Li^+^, La^3+^, and Zr^4+^ sites with dopants can stabilize the cubic phase to enhance its ionic conductivity [12, 13]. For Li‐site doping, metal cations such as Al^3+^, Fe^3+^, and Ga^3+^ have attracted extensive attention [14]. Notably, Al^3+^ has emerged as the preferred dopant on Li‐site for the stabilization of cubic LLZO owing to its excellent doping performance and natural abundance [15, 16, 17].

In addition to cation‐substitution, the doping of anions (e.g., Cl^−^, F^−^) into the garnet structure has been explored to enhance the performance of LLZO [18, 19]. The high electronegativity of the O^2−^ sublattice imparts significant structural rigidity, which consequently suppresses the thermal displacement of the anions [20]. For instance, F^−^ doping promotes the formation of a stable interfacial layer between the electrolyte and the lithium metal anode, thereby enhancing their interfacial compatibility [21]. Moreover, F^−^ substitution in Al‐doped LLZO has been shown to increase the ionic conductivity [22, 23]. Notably, no study has reported nitrogen doping in LLZO, despite successful nitrogen integration elsewhere in solid‐state batteries. Examples include nitrogen‐doped anodic Li oxide films for robust Li metal anodes and solid electrolytes, as well as the use of nitrides to enhance the LLZO/Li anode interface [24, 25]. However, neither the cation doping nor anion doping has improved the ionic conductivity of LLZO substantially (e.g., from approximately 1 × 10^−4^ to 5 × 10^−4^ S cm^−^^1^), and the reasons for the improvement by the doping strategy are not completely understood [26].

In this work, a synergistic cation–anion co‐doping strategy is proposed to optimize Li‐ion transport pathways within the bulk of LLZO via lattice structure modulation. For the first time, Al─N co‐doped LLZO solid electrolytes were successfully synthesized through a facile one‐step solid‐state reaction. The optimized Li_6.00_Al_0.50_La_3_Zr_2_O_11.50_N_0.50_ electrolyte exhibits an exceptional room‐temperature ionic conductivity of 2.19 × 10^−3^ S cm^−1^. The intrinsic mechanisms of the enhanced bulk Li^+^ transport were elucidated through a combination of structural characterizations and DFT simulations, which revealed the pivotal role of the unique local coordination environment in boosting electrolyte performance. Furthermore, the LiFePO_4_/LLZO–Al_0.50_N_0.50_/Li full cell demonstrated superior cycling stability with a capacity retention of 82.60% after 200 cycles, indicating the immense potential of this co‐doping strategy for developing high‐performance all‐solid‐state batteries.

## 2 Results and Discussion

**FIGURE 1:**
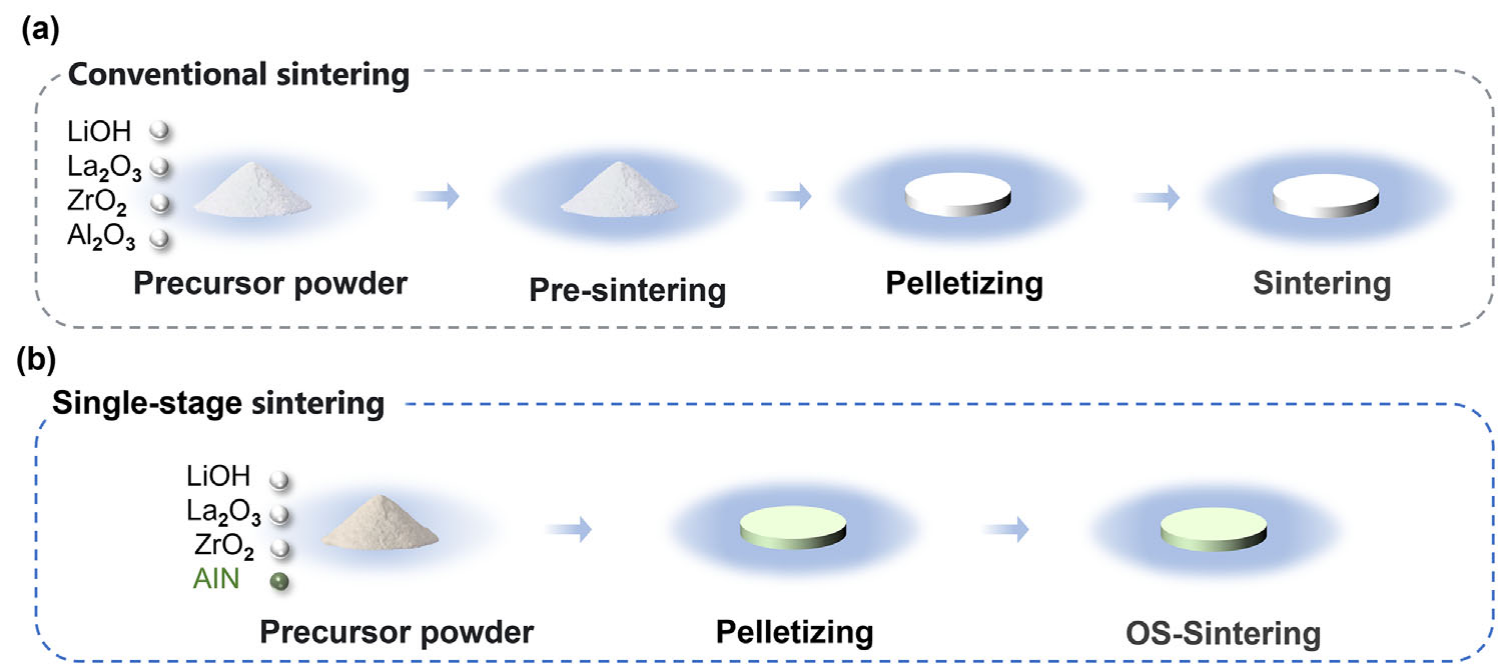
The preparation process of LLZO electrolyte membranes. (a) Conventional preparation and (b) one‐step SSR/sintering.

Figure 1 presents the comparison of conventional solid‐state reaction (SSR) process and one‐step solid‐state reaction/sintering (OS‐SSR) process for LLZO solid electrolytes. As depicted in Figure 1a, the traditional method involves an intermediate SSR, secondary ball‐milling, pellet pressing, and high‐temperature sintering [27, 28]. Such a complex processing procedure inevitably exacerbates lithium volatilization [29]. In contrast, Figure 1b illustrates a streamlined OS‐SSR strategy, wherein the uniformly mixed precursors are directly pressed into pellets for high‐temperature sintering. The one‐step sintering process not only significantly reduces energy consumption and shortens the fabrication cycle, but also mitigates lithium loss, thereby offering a reliable and efficient way for manufacturing high‐quality LLZO solid electrolytes.

**FIGURE 2:**
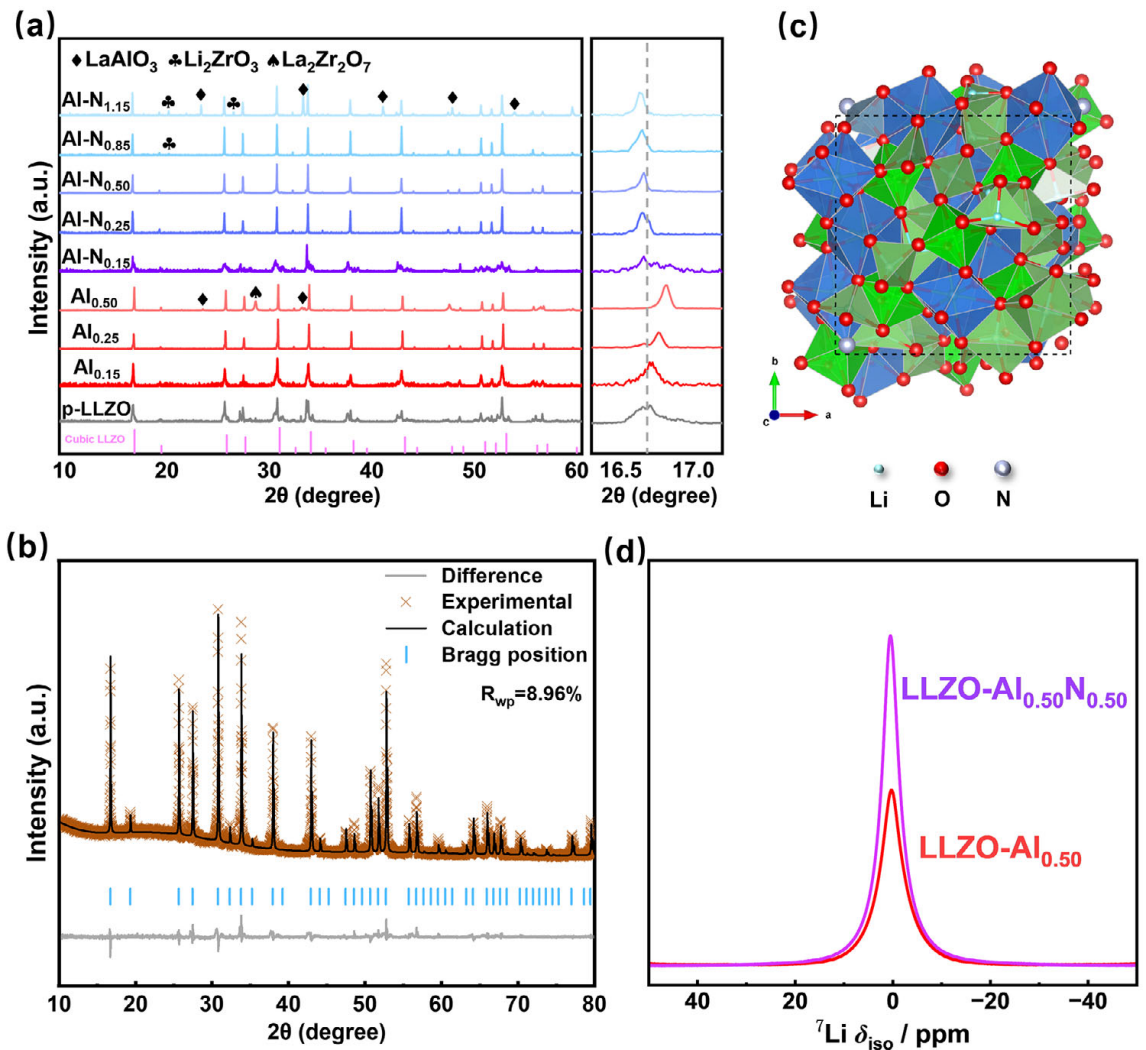
Structural characterization of the modulated SSEs. (a) XRD patterns of the synthesized SSEs. (b) XRD patterns and (c) corresponding Rietveld refinement results of the LLZO–Al_0.50_N_0.50_ electrolyte. (d) ^7^Li NMR spectra of LLZO–Al_0.50_ and LLZO–Al_0.50_N_0.50_.

To systematically investigate the impact of Al─N co‐doping, three distinct sets of electrolytes were prepared via the OS‐SSR process: pristine LLZO (p‐LLZO), Al‐doped LLZO (LLZO–Al*x*, *x* = 0.15, 0.25, 0.50), and Al─N co‐doped LLZO (LLZO‐Al*y*N*y*, *y* = 0.15, 0.25, 0.50, 0.85, 1.15). Their crystal structures were then characterized by X‐ray diffraction (XRD). The XRD patterns reveal that pristine p‐LLZO crystallization forms a tetragonal phase, and Al‐doping and Al─N co‐doping effectively facilitate the phase transition from tetragonal to cubic. Furthermore, the Al─N co‐doped LLZO samples realize a highly pure cubic phase (Figure 2a). Meanwhile, the magnified diffraction pattern on the right side of Figure 2a clearly reveals that, compared to the p‐LLZO, the diffraction peaks of LLZO‐Al_x_ are significantly shifted toward the right, indicating lattice contraction [30]. This contraction is primarily attributed to the substitution of Li^+^ by the smaller Al^3+^, alongside the introduction of lithium vacancies to maintain charge neutrality (3Li^+^ → Al^3+^ + 2V_Li_) [31]. However, after the introduction of N for co‐doping, the diffraction peaks of LLZO–Al_0.50_N_0.50_ show a significant shift toward left compared to the Al‐doped sample, indicating lattice expansion. This subsequent lattice expansion is fundamentally caused by the larger ionic radius of the N^3−^ (0.146 nm) compared to that of O^2−^ (0.14 nm) in the oxygen framework [32, 33].

To further confirm the doping effect, we performed Rietveld refinement of the XRD patterns. The refinement results show that under the same Al doping amount, the lattice constant of Al─N co‐doped LLZO increases to a certain extent (Figure 2b and Figure S1 and Table S1). The lattice constant of LLZO–Al_0.50_N_0.50_ expands to 12.965 Å compared to that of LLZO–Al_0.50_ (12.9537 Å), and the corresponding crystal structure is shown in Figure 2c. Therefore, the XRD results indicate N replaces O in the lattice.

To reveal the local coordination environment of the atoms, solid‐state nuclear magnetic resonance (NMR) measurements were performed. The ^27^Al NMR spectra of LLZO–Al_0.50_ and LLZO–Al_0.50_N_0.50_ exhibit a strong characteristic resonance peak near 70 ppm corresponding to AlO_4_ tetrahedra (Figure S2), indicating that Al^3+^ predominantly occupies the 24d lithium sites in the lattice [34]. Additionally, a minor resonance peak corresponding to AlO_6_ octahedra is observed near 20 ppm, which is attributed to a small fraction of Al^3+^ occupying the 96 h lithium sites. Importantly, the spectral features of the two samples are similar, confirming that the introduction of Al─N co‐doping does not fundamentally alter the primary site occupation preference of Al. Moreover, the ^7^Li NMR spectra (Figure 2d and Table S2) reveal that, compared to the Al‐doped sample, the Li resonance peak of the Al─N co‐doped sample exhibits more pronounced motional narrowing, with its full width at half maximum (FWHM) decreasing from 2.74 to 2.07 ppm. This indicates a higher hopping rate of lithium ions within the lattice, which more effectively averages the local dipole–dipole interactions [35]. This further suggests that the Li─N─O hybrid coordination network derived from N doping reconstructs the local environment of Li, thereby endowing lithium ions with high local mobility.

As the electrolyte synthesis undergoes solid reaction and sintering, the composition of the final electrolyte greatly determines electrolyte properties. To investigate the electrolyte composition, we employed oxygen‐nitrogen‐hydrogen (ONH) analysis and inductively coupled plasma optical emission spectroscopy (ICP‐OES) to measure elements. Firstly, the ONH analysis confirms the incorporation of nitrogen into the co‐doped lattice. Due to the doping limitation, the actual retained N content is approximately 5.2% of the theoretical design value (Table S3). Nevertheless, this critical amount of N doping substantially induce the significant local structural reconstruction [36]. Concurrently, using highly stable La as an internal reference for the ICP‐OES measurements, the lithium amount was measured, and the lithium retention rates were calculated (Figure S3). For solely Al‐doped samples with an identical theoretical doping level, the actual lithium retention rate of the sample prepared by the conventional two‐step sintering method is about 87.3%, while that of the sample prepared by the OS‐SSR method achieves about 90.6%. Notably, when Al─N co‐doping is adopted, the actual lithium retention rate of the OS‐SSR sample further increases to 91.5%. Therefore, the OS‐SSR process and co‐doping strategy effectively alleviate Li loss.

**FIGURE 3:**
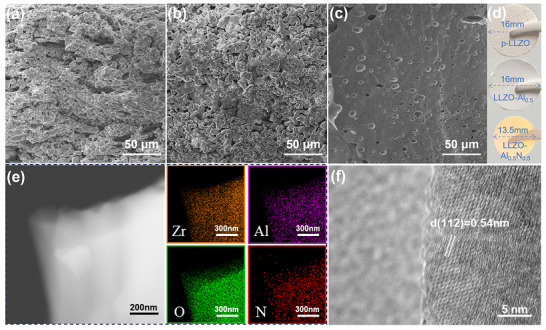
Cross‐sectional SEM images of electrolyte membranes: a) p‐LLZO; b) LLZO–Al_0.50_; c) LLZO–Al_0.50_N_0.50_; d) Digital images of the three pellets; e) TEM image and EDX elemental maps of LLZO–Al_0.50_N_0.50_; f) TEM image of LLZO‐Al_0.50_N_0.50_.

Scanning electron microscopy (SEM) images reveal the high porosity of the p‐LLZO electrolyte (Figure 3a and Figure S4a). The Al‐doped samples show the enhanced sintering, evidenced by particle growth (Figure 3b and Figure S4b,c). Moreover, the Al─N co‐doped LLZO electrolyte shows substantial densification (Figure 3c and Figure S4d–h) with small closed pores. These results explicitly demonstrate that the synergistic co‐doping of Al and N is essential for the single‐step fabrication of highly dense electrolytes. Optical images provide further evidence that the Al─N co‐doped LLZO electrolytes exhibit significant sintering shrinkage according to the pressing die diameter of 16 mm, whereas the p‐LLZO and LLZO–Al_0.50_ samples show almost no shrinkage (Figure 3d and Figure S5). Therefore, these results confirm the Al─N co‐doping improve the sinterability of LLZO, enabling the successful fabrication of highly dense electrolytes under one‐step sintering.

As shown in Figure 3e and Figure S6, energy‐dispersive X‐ray (EDX) mapping images show the uniform distribution of Al and N, confirming the uniform co‐doping of Al and N within LLZO crystals. Additionally, the lattice expansion induced by the Al─N co‐doping was confirmed by transmission electron microscope (TEM). The lattice fringe spacing of 0.54 nm, corresponding to the (112) plane, is slightly larger than that of the p‐LLZO (Figure 3f) [37]. Therefore, Al and N have been successfully doped into the LLZO electrolyte.

**FIGURE 4:**
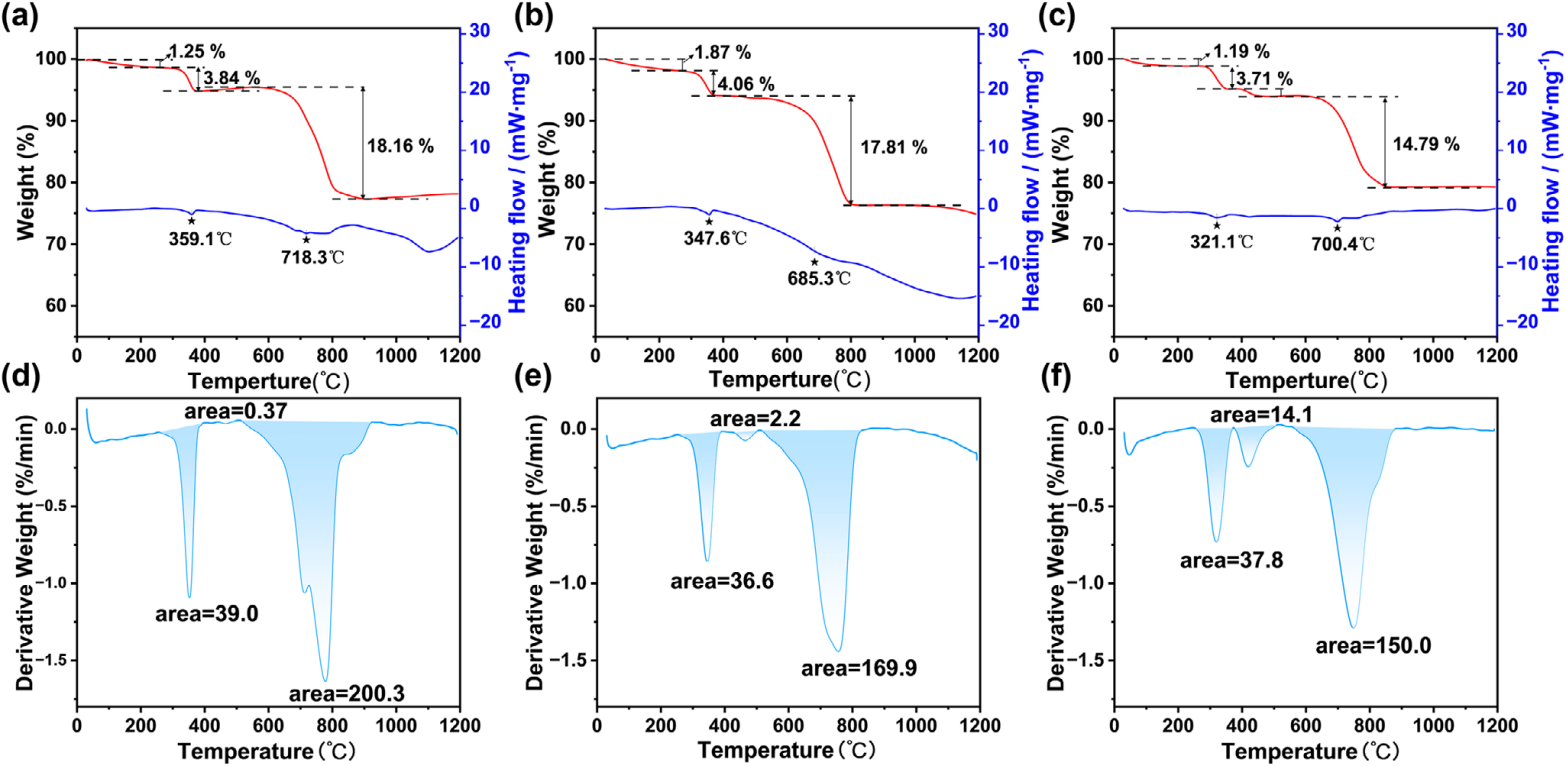
TG/DSC curves of a) p‐LLZO; b) LLZO–Al_0.50_; c) LLZO–Al_0.50_N_0.50_; DTG curves of d) p‐LLZO; e) LLZO–Al_0.50_; f) LLZO–Al_0.50_N_0.50_.

Thermogravimetric‐differential scanning calorimetry (TG‐DSC) was employed to analyze the thermal behavior of the electrolyte precursors. Figure 4a–c shows the TG‐DSC curves of p‐LLZO, LLZO‐Al_0.50_, and LLZO‐Al_0.50_N_0.50_ precursors. Weight loss occurs primarily within three temperature ranges. The first weight loss of 1–2% at temperatures below 250°C is due to the removal of adsorbed moisture. The second weight loss of ≈4% at temperatures between 250 and 500°C is primarily attributed to the dehydration of La_2_O_3_ [38]. The third weight loss at temperatures between 600 and 800°C is 18.18%, 17.81%, and 14.19% for p‐LLZO, LLZO–Al_0.50_ and LLZO–Al_0.50_N_0.50_, respectively. This weight loss is caused by Li_2_CO_3_ decomposition and lithium evaporation [39]. Notably, the weight loss of LLZO–Al_0.50_ initiates at a lower temperature (≈500°C) compared to the other two samples (≈600°C). This is attributed to the catalytic effect of Al_2_O_3_ [40]. As an acidic oxide, Al_2_O_3_ can adsorb CO_3_^2−^, which weakens the C─O bond and lowers the decomposition activation energy, thus catalyzing Li_2_CO_3_ decomposition at the lower temperatures.

The derivative thermogravimetric (DTG) curves in Figure 4d–f further demonstrate the thermal behavior of the electrolytes as peak area is proportional to the mass change [41]. All electrolytes exhibit a major weight‐loss peak within the range of 600–900°C, which corresponds to the third weight loss in the TG‐DSC profiles. The integrated areas of these peaks for p‐LLZO, LLZO–Al_0.50_, and LLZO–Al_0.50_N_0.50_ are 200.3, 169.9, and 150.0, respectively. This indicates that the proper co‐doping strategy fundamentally alters the high‐temperature reaction pathway, effectively mitigating excessive lithium loss during the critical sintering stage.

**FIGURE 5:**
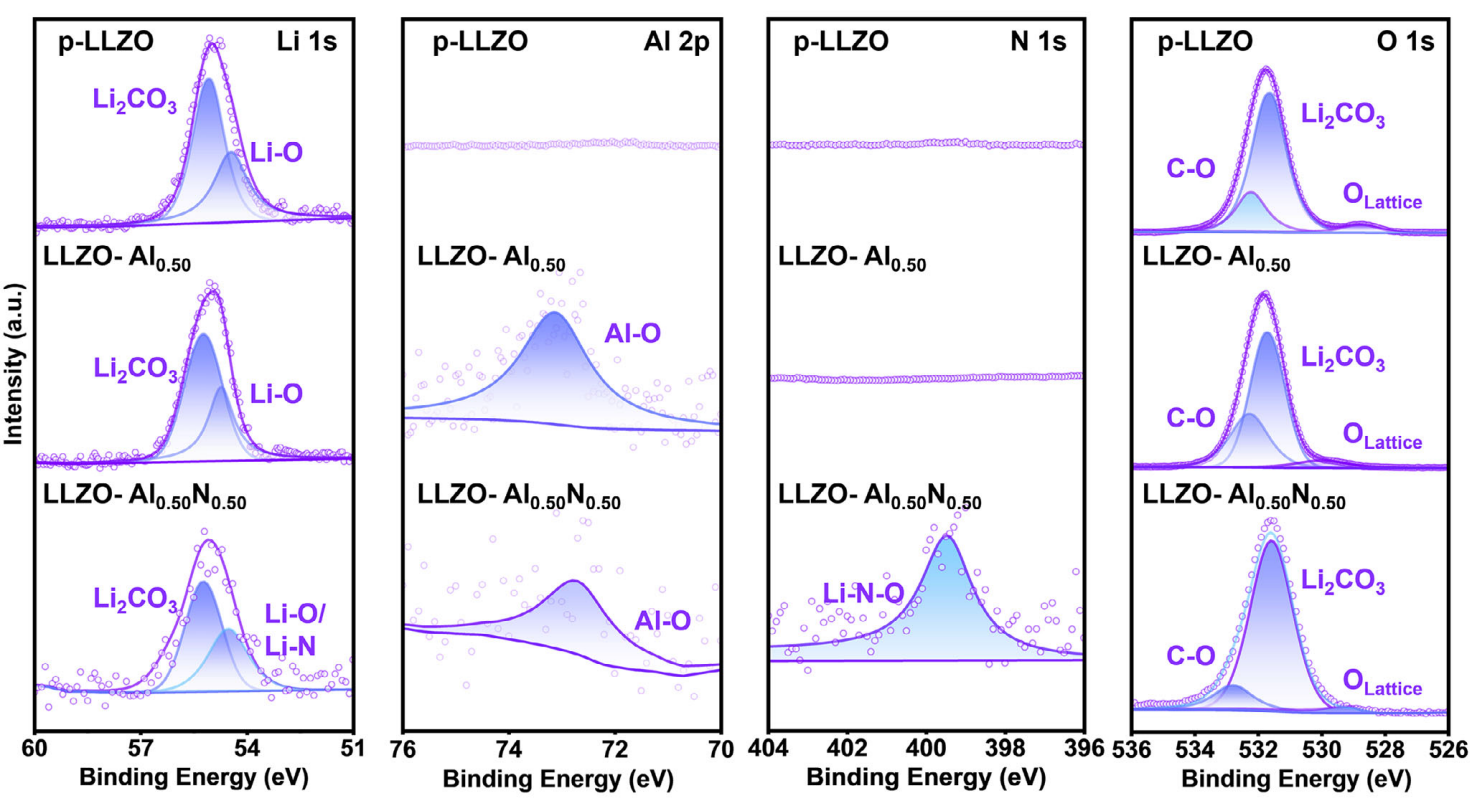
XPS analysis of p‐LLZO, LLZO–Al_0.50_ and LLZO–Al_0.50_N_0.50_.

X‐ray photoelectron spectroscopy (XPS) was employed to systematically investigate the local structural evolution of the solid‐state electrolytes (Figure 5 and Figure S7 and Tables S4–S6). The Li 1s spectra of all samples overlap closely, demonstrating that the fundamental garnet‐type host structure maintains excellent stability and structural integrity [42]. Compared to LLZO–Al_0.50_, the Al 2p spectral peak in LLZO–Al_0.50_N_0.50_ exhibits a distinct broadening and a slight shift toward the lower binding energy, indicating the incorporated nitrogen alters the local chemical coordination environment of Al [43, 44]. Meanwhile, the N 1s XPS spectrum reveals the chemical coordination environment of the incorporated nitrogen. The characteristic peak located at approximately 399.5 eV corresponds to the local Li─N─O coordination network formed by the coexistence of nitrogen and oxygen within the lattice [45].

In addition, the O 1s spectrum further confirms the evolution of the lattice structure and local electronic states. The characteristic lattice oxygen peak of p‐LLZO is located at 528.77 eV. After Al‐doping, this peak shifts to a higher binding energy of 530.01 eV. This shift is primarily attributed to the stronger electron‐attracting ability of Al compared to Li, which reduces the electron cloud density around the adjacent oxygen atoms [46]. Notably, for Al─N co‐doping, the lattice oxygen peak position shifts back to a lower binding energy of 529.14 eV. This is mainly because the electronegativity of nitrogen (3.04) is less than that of oxygen (3.44) [47]. After N^3−^ is integrated into the lattice, it plays a compensatory role for the local electron density, enhancing the extranuclear electron shielding effect of the remaining oxygen atoms [45]. This evolution of binding energy proves that Al and N elements have successfully co‐doped into the garnet framework.

**FIGURE 6:**
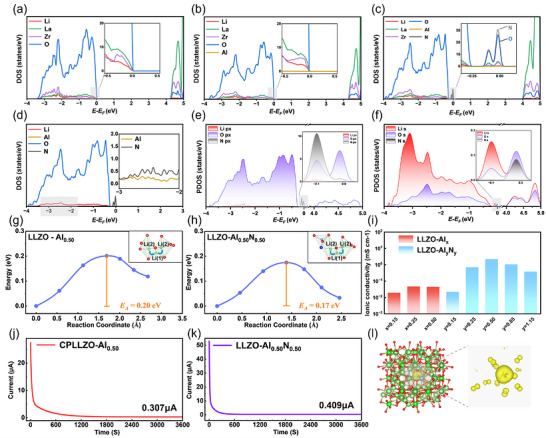
The calculated density of states (DOS) of a) p‐LLZO, b) LLZO–Al_0.50_, c) LLZO‐Al_0.50_N_0.50_; d) The calculated DOS of Li, Al, O and N in LLZO‐Al_0.50_N_0.50_; The calculated partial density of states (PDOS) of different electron orbitals of LLZO‐Al_0.50_N_0.50_ of e) p orbitals and f) s orbitals. The activation energy of Li^+^ migration from Li(2) site to neighboring Li(2) site of g) LLZO–Al_0.50_ and h) LLZO–Al_0.50_N_0.50_. i) Ionic conductivity of LLZO–Al_x_ and LLZO–Al_y_N_y_ at 25°C. DC polarization curves of (j) CPLLZO–Al_0.50_ and (k) LLZO–Al_0.50_N_0.50_. (l) Partial charge density of electronic states near the Fermi level for LLZO–Al_0.50_N_0.50_.

To gain fundamental insights into the N substitution at O sites within the LiO_4_ tetrahedra (Figure S8), the electronic density of states (DOS) of the electrolytes was systematically investigated using density functional theory (DFT) [48]. As shown in Figure 6a–c, LLZO–Al_0.50_N_0.50_ exhibits a distinct electronic structure compared to p‐LLZO and LLZO–Al_0.50_. The DOS reveals the emergence of new localized electronic states near the Fermi level [49]. Furthermore, LLZO–Al_0.50_N_0.50_ displays overlapping contributions from N and O orbitals in these new states, which unequivocally confirms the successful substitution of O by N within the lattice. In the ‐4 to 0.3 eV range, the broad overlapping peaks of the Al and N density of states (Figure 6d) indicate that the Al─N bond possesses the lower polarity and stronger covalency compared to the Al─O bond [50]. More importantly, the high degree of overlapping among the s and p orbitals of Li, N, and O near the Fermi level (Figure 6e,f) signifies strong orbital hybridization among these three elements, further confirming the formation of a local Li─N─O coordination structure [51].

To investigate the effect of Al─N co‐doping on lithium transport kinetics, we performed nudged elastic band (NEB) calculations. Figure 6g,h displays the activation energy of Li^+^ migration from the tetrahedral Li(1) site to the octahedral Li(2) site [52, 53], yielding the values of 0.20 and 0.17 eV for the Al‐doped LLZO and Al─N co‐doped LLZO, respectively. The reduction in the migration energy barrier indicates that N doping creates a more favorable energy landscape for Li^+^ transport [30]. The origin of this reduction can be further rationalized using Fajans' rules and the hard‐soft acid‐base (HSAB) theory [54]. Since the Pauling electronegativity of N is lower than that of O, the local bonds involving N exhibit more covalent character than the highly ionic Li─O bonds [55]. This is quantitatively supported by Bader charge analysis. Specifically, the net charge of the N dopant is calculated to be –1.666 |e|, representing a deviation of 1.334 |e| from its formal oxidation state (–3). This deviation is significantly larger than that of the average O atom in the lattice (approximately 0.759 |e|). This indicates that the N atom does not fully withdraw electrons but instead extensively shares its electron cloud highly with the surrounding atoms. Furthermore, the positive charge of Li atoms adjacent to the N doping center (+0.394 |e|) is also notably lower than the average value of Li atoms in the lattice (+0.436 |e|). This further confirms that the electron cloud distribution between Li and N is more uniform compared to that of Li–O, exhibiting stronger covalent interactions that weaken the electrostatic restriction on Li^+^ ions. From the perspective of the HSAB framework, Li^+^ acts as a “hard acid” and O^2^^−^ as a “hard base,” which typically form a rigid electrostatic network that restricts ion migration [56, 57]. The introduction of the N dopant creates a more flexible local coordination environment. The highly polarizable electron cloud of N can undergo dynamic deformation to effectively stabilize the cation in the transition state [54]. This dynamic interaction greatly smooths the potential energy surface, fundamentally explaining the significant reduction in ion transport activation energy.

In addition, electrochemical impedance spectroscopy (EIS) was employed to measure the ionic conductivity of the samples and perform equivalent circuit fitting. Compared to the Al‐doped, the Al─N co‐doped LLZO electrolyte exhibits significantly reduced bulk resistance (*R*_bulk_) and substantially enhanced total ionic conductivity (Figure 6i and Figure S9 and Table S7). In addition, to further exclude the contribution of densification induced by Al─N co‐doping to the enhancement of ionic conductivity, a highly dense Al‐doped control sample (CPLLZO‐Al_x_) was prepared using a conventional sintering process (Figures S10 and S11 and Table S7). Although the relative density of CPLLZO–Al_0.50_ reached 93.4%, the intrinsic *R*_bulk_ of LLZO–Al_0.50_N_0.50_ remained significantly lower than that of CPLLZO–Al_0.50_. Consequently, the total ionic conductivity of LLZO–Al_0.50_N_0.50_ (2.19 × 10^−3^ S cm^−1^) remained nearly one order of magnitude higher than that of the highly dense CPLLZO–Al_0.50_ (1.36 × 10^−4^ S cm^−1^). To confirm the synergistic co‐doping enhances the ionic conductivity of LLZO, we attempted to synthesize nitrogen‐doped LLZO samples. XRD results show that, except for some La_2_Zr_2_O_7_ impurity phases, the samples exhibit a cubic phase structure (Figure S12). Furthermore, all nitrogen‐doped LLZO samples exhibit ionic conductivities with the order of 10^−5^ S cm^−1^ (Figure S13b and Table S7), which is significantly lower than those of Al─N co‐doped samples. The results indicate nitrogen doping leads to only a limited enhancement of the ionic conductivity of LLZO. This comparative analysis unequivocally confirms that the superior ionic conductivity of LLZO–Al*_y_* N*_y_* is fundamentally attributed to the optimization of the internal transport environment within the bulk lattice via Al─N co‐doping, rather than merely to densification.

Based on the above analysis, we further explored the temperature‐dependent ion transport kinetics. The apparent activation energies (*E*a) of the electrolytes were determined by linearly fitting the Arrhenius plots (Figure S14a) [58]. Consistent with the ionic conductivity results, the activation energies of the LLZO–Al*_y_* N*_y_* electrolytes are lower than those of the LLZO–Al_x_ electrolytes, with LLZO–Al_0.50_N_0.50_ exhibiting the lowest value of 0.26 eV (Figure S14b). It should be noted that the experimentally measured macroscopic *E*a is numerically higher than the NEB‐calculated microscopic barrier (0.17 eV), primarily because the macroscopic *E*a reflects the long‐range percolation of ions at finite temperatures, which inevitably entails additional energy penalties. Despite this numerical discrepancy rooted in different physical regimes, the lower migration barrier and activation energy of LLZO–Al_0.50_N_0.50_ strongly corroborate that Al─N co‐doping effectively reduces the intrinsic kinetic barrier for Li^+^ conduction, thereby enhancing the ionic conductivity.

In addition, the macroscopic electronic conductivity (σ_e_) of the samples was evaluated using the DC polarization (Hebb‐Wagner) method. The resulting chronoamperometry profiles (Figure 6j,k and Figure S15) demonstrate that the steady‐state σ_e_ of the Al─N co‐doped LLZO–Al_0.50_N_0.50_ electrolyte remains at an extremely low level, which is highly comparable to that of Al‐doped LLZO prepared by conventional sintering. Compared to its ionic conductivity, the electronic contribution is almost negligible (Tables S7 and S8). Meanwhile, to further elucidate the nature of the newly introduced electronic states near the Fermi level from a microscopic perspective, we calculated the partial charge density corresponding to these defect states. As illustrated in Figure 6l, the electron density of these newly introduced states is spatially confined strictly to the region of the N dopant and its immediately adjacent atoms. No continuous or delocalized electron cloud distribution is observed in the lattice, confirming that these new states are highly localized and defect‐related. Because these localized defect states are spatially isolated from one another, it cannot form a percolation pathway required for electronic conduction [59]. Collectively, the experimental measurements and theoretical calculations confirm that Al─N co‐doping does not compromise the intrinsic electronic insulating properties of LLZO, thereby eliminating the risk of electronic leakage and the subsequent growth of lithium dendrites.

Combining the comprehensive findings and theoretical analysis of this study, the fundamental reasons of the high ionic conductivity in Al─N co‐doped LLZO were further elucidated by comparing our work with recently reported advanced doping systems (Table S9). From the fundamental perspective of defect equilibrium, conventional single‐cation doping (e.g., Al^3+^, Ga^3+^) stabilizes the cubic phase but inevitably generates a large amount of lithium vacancies to maintain electroneutrality, leading to a reduction in active Li^+^ carriers [14, 16]. In Al─F co‐doping, the highly electronegative F^−^ (3.98) exerts a strong electrostatic attraction on Li^+^, inadvertently acting as a local “energy trap” that hinders long‐range ion hopping [22]. Conversely, N possesses a relatively lower electronegativity than both O and F, coupled with a higher anion polarizability [60]. This unique local Li─N─O coordination effectively softens the electrostatic restraint of the rigid anion framework on Li^+^, significantly lowering the intrinsic migration barrier, thereby accelerating Li^+^ transport. Therefore, the synergistic optimization of rapid ion transport channels in the bulk phase by Al─N co‐doping is key to the exceptional total ionic conductivity of this system.

**FIGURE 7:**
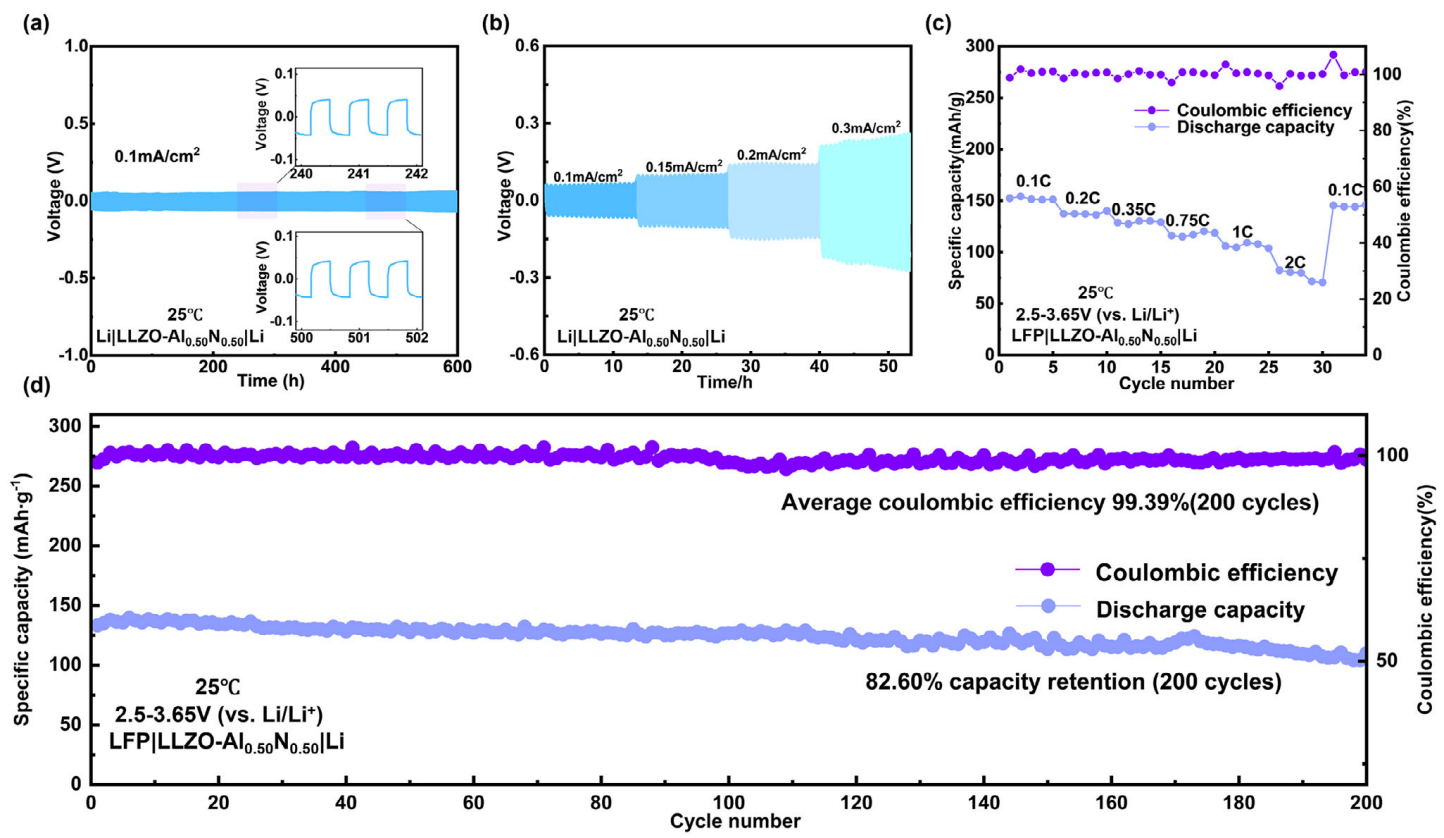
Galvanostatic cycling test of the symmetric Li/LLZO–Al_0.50_N_0.50_/Li cell at a) 0.1 mA cm^−2^ and b) different current densities. c) Rate performance of the LFP/LLZO–Al_0.50_N_0.50_/Li full cell. d) Cyclic stability of LFP/LLZO–Al_0.50_N_0.50_/Li full cell at 0.3C.

To evaluate the electrochemical stability of LLZO–Al_y_N_y_ against lithium metal, we employed a molten lithium process to assemble Li|LLZO–Al_y_N_y_|Li symmetric cells and tested their lithium stripping/plating cycling performance. As shown in Figure 7a, the symmetric cell achieved an ultra‐long stable cycling life of over 600 h at a current density of 0.1 mA cm^−2^. In stark contrast, under the same testing conditions, the Al‐doped LLZO–Al_0.50_ (Figure S16) exhibited severe voltage fluctuations and subsequent short‐circuiting after only 120 h of cycling, along with a significantly higher polarization voltage. Meanwhile, the interfacial stability of the post‐cycled LLZO–Al_0.50_N_0.50_ electrolyte surface was investigated. The sample surface exhibits a highly dense and intact morphology without cracks or lithium dendrite penetration (Figure S17), indicating a mechanically robust interface that effectively suppresses uneven lithium plating. XPS analysis confirms its exceptional chemical stability (Figure S18). The intrinsic chemical framework remains well‐preserved with an intact Li─N─O coordination structure, confirming the Al─N co‐doped lattice is not destructively reduced by the highly reactive lithium metal during long‐term cycling. Accordingly, the intact morphology and stable interfacial state firmly demonstrate the superior structural and electrochemical stability of the Al─N co‐doped electrolyte against the lithium metal anode. Figure S19 displays the critical current density of the LLZO–Al_0.50_N_0.50_ electrolyte, which is 1.0 mA cm^−2^, highlighting its excellent resistance to lithium dendrite penetration. To further explore the compatibility of LLZO–Al_0.50_N_0.50_ in a full‐cell configuration, we assembled hybrid‐configuration full cells using LiFePO_4_ as the cathode. As shown in Figure 7c and Figure S20, this cell exhibited excellent rate capability. At rates of 0.1, 0.2, 0.35, 0.75, 1, and 2 C, the average specific discharge capacities were 152.8, 137.6, 129.2, 117.4, 106.3, and 77.0 mAh g^−1^, respectively. When the current density was restored to 0.1 C, the discharge capacity recovered to 145.4 mAh g^−1^, demonstrating good rate performance. In subsequent long‐term cycling stability tests, the LFP/LLZO–Al_0.50_N_0.50_/Li cell operated stably for over 200 cycles at a rate of 0.3 C, with an average Coulombic efficiency of 99.39% throughout the cycling process.

## 3 Conclusion

Utilizing a one‐step sintering process, we successfully synthesized Al–N co‐doped LLZO, which resulted in a highly dense solid electrolyte with enhanced ionic conductivity. The Al─N co‐doping significantly improved both the sinterability and the ionic conductivity of LLZO. The synergistic cation–anion co‐doping strategy significantly enhanced the ionic transport properties compared to single Al‐doping, effectively optimizing the lattice environment for rapid Li‐ion conduction. The optimized LLZO–Al_0.50_N_0.50_ electrolyte achieved a remarkable room‐temperature ionic conductivity of 2.19 × 10^−3^ S cm^−1^. NEB calculations further elucidated that the incorporation of nitrogen effectively reduces the activation energy for Li^+^ migration by modulating the migration pathways. Beyond its superior bulk conductivity, the electrolyte demonstrated exceptional electrochemical stability and interfacial compatibility with lithium metal anodes. The as‐assembled LiFePO_4_/LLZO–Al_0.50_N_0.50_/Li full cell maintained a high‐capacity retention of 82.6% after 200 cycles at room temperature. These findings underscore the immense potential of the cation–anion co‐doping strategy as a pivotal approach for advancing the practical realization of high‐performance all‐solid‐state lithium batteries.

## Author Contributions

**Jiaming Wu**: methodology, writing – review and editing. **Jiajie Li**: conceptualization, investigation, funding acquisition, writing – original draft, writing – review and editing, supervision, project administration. **Yaocong Wang**: methodology, software, validation, writing – review and editing, formal analysis. **Xundao Liu**: writing – review and editing. **Hao Zhang**: methodology, software, data curation, investigation, formal analysis, validation, visualization, writing – review and editing. **Quande Che**: software, writing – review and editing, formal analysis, data curation. **Dongxu Mao**: methodology, investigation, writing – review and editing. **Dehua Dong**: conceptualization, writing – review and editing, supervision, resources, funding acquisition. **Yanzhu Zhang**: software, formal analysis, writing – review and editing. **Zhengmao Ye**: writing – review and editing, resources, supervision, funding acquisition.

## Conflicts of Interest

The authors declare no conflicts of interest.

## Supporting information




**Supporting File**: advs75980‐sup‐0001‐SuppMat.docx.

## Data Availability

The data that support the findings of this study are available from the corresponding author upon reasonable request.

## References

[1] M. Li , J. Lu , Z. Chen , and K. Amine , “30 Years of Lithium‐Ion Batteries,” Advanced Materials 30 (2018): 1800561. 10.1002/adma.201800561 29904941

[2] P. Jaumaux , J. Wu , D. Shanmukaraj , et al., “Non‐Flammable Liquid and Quasi‐Solid Electrolytes toward Highly‐Safe Alkali Metal‐Based Batteries,” Advanced Functional Materials 31 (2021): 2008644.

[3] R. Gond , W. van Ekeren , R. Mogensen , A. J. Naylor , and R. Younesi , “Non‐Flammable Liquid Electrolytes for Safe Batteries,” Materials Horizons 8 (2021): 2913–2928. 34549211 10.1039/d1mh00748c

[4] S. Yang , W. Xin , X. Liu , et al., “In‐Situ Preparation of Tightly Contacting 3D fiber Network Composite Electrolytes for High‐Performance All‐Solid‐State Lithium Batteries,” Journal of Electroanalytical Chemistry 979 (2025): 118915.

[5] M. Zhang , Y. He , Z. Li , et al., “Data‐Driven Ionization‐Energy Descriptor Enables Stable Cathode‐Electrolyte Interface in All‐Solid‐State Sodium‐Metal Batteries,” Advanced Functional Materials 36 (2026): 27652.

[6] Z. Fang , M. Zhang , Z. Zhang , et al., “In Situ Constructing Robust Interface by Deep Eutectic Polymeric Electrolyte Enables High Performance Lithium Metal Batteries with High‐Loading Cathode,” Advanced Science 11 (2024): 2411421. 39465907 10.1002/advs.202411421 PMC11653719

[7] Q. Zhao , S. Stalin , C.‐Z. Zhao , and L. A. Archer , “Designing Solid‐State Electrolytes for Safe, Energy‐Dense Batteries,” Nature Reviews Materials 5 (2020): 229–252.

[8] M. Zhang , Y. He , J. Li , et al., “Bandgap‐Preserving co‐Doping Strategy for NASICON Electrolytes with Concurrent Optimization of Ionic and Electronic Transport,” Energy Storage Materials 81 (2025): 104547.

[9] H. Sun , S. Kang , and L. Cui , “Prospects of LLZO Type Solid Electrolyte: from Material Design to Battery Application,” Chemical Engineering Journal 454 (2023): 140375.

[10] Y. Wang , Z. Chen , K. Jiang , Z. Shen , S. Passerini , and M. Chen , “Accelerating the Development of LLZO in Solid‐State Batteries Toward Commercialization: A Comprehensive Review,” Small 20 (2024): 2402035. 10.1002/smll.202402035 38770746

[11] Y. You , D. Zhang , X. Cao , T.‐Y. Lü , Z.‐Z. Zhu , and S. Wu , “Exploring High‐Valence Element Doping in LLZO Electrolytes: Effects on Phase Transition and Lithium‐Ion Conductivity,” Journal of Power Sources 612 (2024): 234831.

[12] A. Kim , J.‐H. Kang , K. Song , and B. Kang , “Simultaneously Improved Cubic Phase Stability and Li‐Ion Conductivity in Garnet‐Type Solid Electrolytes Enabled by Controlling the Al Occupation Sites,” ACS Applied Materials & Interfaces 14 (2022): 12331–12339. 35213140 10.1021/acsami.2c01361

[13] L. Dhivya and R. Murugan , “Effect of Simultaneous Substitution of Y and Ta on the Stabilization of Cubic Phase, Microstructure, and Li^+^ Conductivity of Li_7_La_3_Zr_2_O_12_ Lithium Garnet,” ACS Applied Materials & Interfaces 6 (2014): 17606–17615. 25265573 10.1021/am503731h

[14] X. Xiang , Y. Liu , F. Chen , et al., “Crystal Structure and Lithium Ionic Transport Behavior of Li Site DopedLi_7_La_3_Zr_2_O_12_ ,” Journal of the European Ceramic Society 40 (2020): 3065–3071.

[15] T. Thompson , J. Wolfenstine , J. L. Allen , et al., “Tetragonal vs. Cubic Phase Stability in Al–Free Ta Doped Li_7_La_3_Zr_2_O_12_ (LLZO),” Journal of Materials Chemistry A 2 (2014): 13431–13436.

[16] C. Im , D. Park , H. Kim , and J. Lee , “Al‐Incorporation into Li_7_La_3_Zr_2_O_12_ Solid Electrolyte Keeping Stabilized Cubic Phase for All‐Solid‐State Li Batteries,” Journal of Energy Chemistry 27 (2018): 1501–1508.

[17] M. R. Bonilla , F. A. G. Daza , J. Carrasco , and E. Akhmatskaya , “Exploring Li‐Ion Conductivity in Cubic, Tetragonal and Mixed‐Phase Al‐Substituted Li_7_La_3_Zr_2_O_12_ Using Atomistic Simulations and Effective Medium Theory,” Acta Materialia 175 (2019): 426–435.

[18] B. Dong , A. R. Haworth , S. R. Yeandel , et al., “Halogenation of Li_7_La_3_Zr_2_O_12_ Solid Electrolytes: a Combined Solid‐State NMR, Computational and Electrochemical Study,” Journal of Materials Chemistry A 10 (2022): 11172–11185.

[19] R. Pfenninger , M. Struzik , I. Garbayo , E. Stilp , and J. L. Rupp , “A Low Ride on Processing Temperature for Fast Lithium Conduction in Garnet Solid‐State Battery Films,” Nature Energy 4 (2019): 475–483.

[20] Y. Lu , X. Meng , J. A. Alonso , M. T. Fernández‐Díaz , and C. Sun , “Effects of Fluorine Doping on Structural and Electrochemical Properties of Li_6.25_Ga_0.25_La_3_Zr_2_O_12_ as Electrolytes for Solid‐State Lithium Batteries,” ACS Applied Materials & Interfaces 11 (2018): 2042–2049. 30562455 10.1021/acsami.8b17656

[21] J. Biao , B. Han , Y. Cao , et al., “Inhibiting Formation and Reduction of Li_2_CO_3_ to LiC_X_ at Grain Boundaries in Garnet Electrolytes to Prevent Li Penetration,” Advanced Materials 35 (2023): 2208951. 10.1002/adma.202208951 36639140

[22] X. Ma and Y. Xu , “Efficient Anion Fluoride‐Doping Strategy to Enhance the Performance in Garnet‐Type Solid Electrolyte Li_7_La_3_Zr_2_O_12_ ,” ACS Applied Materials & Interfaces 14 (2022): 2939–2948. 34991309 10.1021/acsami.1c21951

[23] Y. Yang and H. Zhu , “Effects of F and Cl Doping in Cubic Li_7_La_3_Zr_2_O_12_ Solid Electrolyte: A First‐Principles Investigation,” ACS Applied Energy Materials 5 (2022): 15086–15092.

[24] C. Zhang , X. Hu , Z. Nie , et al., “High‐Performance Ta‐Doped Li_7_La_3_Zr_2_O_12_ Garnet Oxides with AlN Additive,” Journal of Advanced Ceramics 11 (2022): 1530–1541.

[25] X. Kan , X. Chang , K. Liu , M. Jia , Z. Bi , and X. Guo , “Implanting Non‐Lithiated Metal into Fluoride/Nitride Hybrid Matrix Enabling Tailored Lithium/Garnet Interface Chemistry for Durable Solid‐State Lithium Batteries,” Advanced Functional Materials 36 (2025): 12506.

[26] A. Sodhiya , A. K. Singh , S. Soni , S. Patel , and R. Kumar , “Structural and Transport Properties Study of Fluorine Doped Li_6.4_Al_0.2_La_3_Zr_2_O_12_ Electrolyte,” Applied Physics A 128 (2022): 639.

[27] V. Thangadurai , S. Narayanan , and D. Pinzaru , “Garnet‐Type Solid‐State Fast Li Ion Conductors for Li Batteries: Critical Review,” Chemical Society Reviews 43 (2014): 4714–4727. 24681593 10.1039/c4cs00020j

[28] K. J. Kim , M. Balaish , M. Wadaguchi , L. Kong , and J. L. Rupp , “Solid‐State Li–Metal Batteries: Challenges and Horizons of Oxide and Sulfide Solid Electrolytes and Their Interfaces,” Advanced Energy Materials 11 (2021): 2002689.

[29] J. Wolfenstine , E. Rangasamy , J. L. Allen , and J. Sakamoto , “High Conductivity of Dense Tetragonal Li_7_La_3_Zr_2_O_12_ ,” Journal of Power Sources 208 (2012): 193–196.

[30] A. C. Moy , A. Manjón‐Sanz , T. C. Caracciolo , M. V. Lobanov , G. M. Veith , and J. Sakamoto , “Effects of Al Concentration on the Structure and Conductivity of Lithium Lanthanum Zirconium Oxide,” Journal of Materials Chemistry A 12 (2024): 28193–28210.

[31] A. G. Squires , D. O. Scanlon , and B. J. Morgan , “Native Defects and Their Doping Response in the Lithium Solid Electrolyte Li_7_La_3_Zr_2_O_12_ ,” Chemistry of Materials 32 (2019): 1876–1886.

[32] R. D. Shannon , “Revised Effective Ionic Radii and Systematic Studies of Interatomic Distances in Halides and Chalcogenides”Foundations of Crystallography 32 (1976): 751–767.

[33] I. Marozau , A. Shkabko , G. Dinescu , et al., “Pulsed laser deposition and characterization of nitrogen‐substituted SrTiO_3_ thin films,” Applied Surface Science 255 (2009): 5252–5255.

[34] B. Karasulu , S. P. Emge , M. F. Groh , C. P. Grey , and A. J. Morris , “Al/Ga‐Doped Li_7_La_3_Zr_2_O_12_ Garnets as Li‐Ion Solid‐State Battery Electrolytes: Atomistic Insights into Local Coordination Environments and Their Influence on 17 O, 27 Al, and 71 Ga NMR Spectra,” Journal of the American Chemical Society 142 (2020): 3132–3148. 31951131 10.1021/jacs.9b12685 PMC7146863

[35] A. Kuhn , S. Narayanan , L. Spencer , G. Goward , V. Thangadurai , and M. Wilkening , “Li Self‐Diffusion in Garnet‐Type Li_7_La_3_Zr_2_O_12_ as Probed Directly by Diffusion‐Induced Li7 Spin‐Lattice Relaxation NMR Spectroscopy,” Physical Review B 83 (2011): 094302.

[36] J.‐N. Zhang , Q. Li , C. Ouyang , et al., “Trace Doping of Multiple Elements Enables Stable Battery Cycling of LiCoO_2_ at 4.6 V,” Nature Energy 4 (2019): 594–603.

[37] P. Jiang , Y. Shi , K. Li , et al., “Research Progress of Key Materials for Aluminum‐Ion Batteries,” Energy Storage Science and Technology 9 (2020): 523.

[38] R. P. Rao , W. Gu , N. Sharma , V. K. Peterson , M. Avdeev , and S. Adams , “In Situ Neutron Diffraction Monitoring of Li_7_La_3_Zr_2_O_12_ Formation: toward a Rational Synthesis of Garnet Solid Electrolytes,” Chemistry of Materials 27 (2015): 2903–2910.

[39] M. Rosen , R. Ye , M. Mann , et al., “Controlling the Lithium Proton Exchange of LLZO to Enable Reproducible Processing and Performance Optimization,” Journal of Materials Chemistry A 9 (2021): 4831–4840.

[40] R. A. de Oliveira , A. L. da Silva , L. B. Caliman , and D. Gouvea , “Interface Excess on Li_2_O‐Doped γ‐Al_2_O_3_ Nanoparticles,” Ceramics International 46 (2020): 10555–10560.

[41] P. Ptáček , F. Šoukal , T. Opravil , J. Havlica , and J. Brandštetr , “The Kinetic Analysis of the Thermal Decomposition of Kaolinite by DTG Technique,” Powder Technology 208 (2011): 20–25.

[42] Y. Zhu , J. G. Connell , S. Tepavcevic , et al., “Dopant‐Dependent Stability of Garnet Solid Electrolyte Interfaces with Lithium Metal,” Advanced Energy Materials 9 (2019): 1803440.

[43] J. Bates , N. Dudney , G. Gruzalski , et al., “Fabrication and Characterization of Amorphous Lithium Electrolyte Thin Films and Rechargeable Thin‐Film Batteries,” Journal of Power Sources 43 (1993): 103–110.

[44] M. Chen , X. Wang , Y. Yu , et al., “X‐ray Photoelectron Spectroscopy and Auger Electron Spectroscopy Studies of Al‐Doped ZnO Films,” Applied Surface Science 158 (2000): 134–140.

[45] P. S. Bagus , E. S. Ilton , and C. J. Nelin , “The Interpretation of XPS Spectra: Insights into Materials Properties,” Surface Science Reports 68 (2013): 273–304.

[46] L. Pauling , “The Nature of the Chemical Bond—1992,” Journal of Chemical Education 69 (1992): 519.

[47] A. L. Allred , “Electronegativity Values from Thermochemical Data,” Journal of Inorganic and Nuclear Chemistry 17 (1961): 215–221.

[48] H. U. Lee , S. Han , D. G. Lee , et al., “Isovalent Multi‐Component Doping Strategy for Stabilizing Cubic‐Li_7_La_3_Zr_2_O_12_ with Excellent Li Mobility,” Chemical Engineering Journal 471 (2023): 144552.

[49] A. Moradabadi and P. Kaghazchi , “Defect Chemistry in Cubic Li_6.25_Al_0.25_La_3_Zr_2_O_12_ Solid Electrolyte: A Density Functional Theory Study,” Solid State Ionics 338 (2019): 74–79.

[50] S. Zhang , F. Zhao , L. Li , and X. Sun , “Solid‐State Electrolytes Expediting Interface‐Compatible Dual‐Conductive Cathodes for All‐Solid‐State Batteries,” Energy & Environmental Science 18 (2025): 6530–6539.

[51] H.‐K. Tian , B. Xu , and Y. Qi , “Computational Study of Lithium Nucleation Tendency in Li_7_La_3_Zr_2_O_12_ (LLZO) and Rational Design of Interlayer Materials to Prevent Lithium Dendrites,” Journal of Power Sources 392 (2018): 79–86.

[52] M. Xu , M. S. Park , J. M. Lee , T. Y. Kim , Y. S. Park , and E. Ma , “Mechanisms of Li^+^ Transport in Garnet‐Type Cubic Li_3+X_ La_3_M_2_O_12_ ( M = Te, Nb, Zr),” Physical Review B 85 (2012): 052301.

[53] Y. Zhang , F. Chen , J. Li , et al., “Regulation Mechanism of Bottleneck Size on Li^+^ Migration Activation Energy in Garnet‐Type Li_7_La_3_Zr_2_O_12_ ,” Electrochimica Acta 261 (2018): 137–142.

[54] M. A. Kraft , S. P. Culver , M. Calderon , et al., “Influence of Lattice Polarizability on the Ionic Conductivity in the Lithium Superionic Argyrodites Li_6_PS_5_ X (X = Cl, Br, I),” Journal of the American Chemical Society 139 (2017): 10909–10918. 28741936 10.1021/jacs.7b06327

[55] K. Pitzer , “The Nature of the Chemical Bond and the Structure of Molecules and Crystals: An Introduction to Modern Structural Chemistry,” Journal of the American Chemical Society 82 (1960): 4121–4121.

[56] J. C. Bachman , S. Muy , A. Grimaud , et al., “Inorganic Solid‐State Electrolytes for Lithium Batteries: Mechanisms and Properties Governing Ion Conduction,” Chemical Reviews 116 (2016): 140–162. 26713396 10.1021/acs.chemrev.5b00563

[57] R. G. Pearson , “Hard and Soft Acids and Bases,” Journal of the American Chemical Society 85 (1963): 3533–3539.

[58] J. Awaka , N. Kijima , H. Hayakawa , and J. Akimoto , “Synthesis and Structure Analysis of Tetragonal Li_7_La_3_Zr_2_O_12_ with the Garnet‐Related Type Structure,” Journal of Solid State Chemistry 182 (2009): 2046–2052.

[59] F. Han , A. S. Westover , J. Yue , et al., “High Electronic Conductivity as the Origin of Lithium Dendrite Formation within Solid Electrolytes,” Nature Energy 4 (2019): 187–196.

[60] A. Fuertes , “Chemistry and Applications of Oxynitride Perovskites,” Journal of Materials Chemistry 22 (2012): 3293–3299.

