# Supplementary material for: Al─N Co‐Doped LLZO Solid Electrolytes via One‐Step Sintering: Toward High Ionic Conductivity
Source: Adv Sci (Weinh). 2026 Jun 9:e75980. Online ahead of print. doi: 10.1002/advs.75980 (PMC13336599; doi:10.1002/advs.75980)
Supplement: Supplementary file 1 — Supporting File: advs75980‐sup‐0001‐SuppMat.docx. [file ADVS-9999-e75980-s001.docx]

Al-N Co-doped LLZO Solid Electrolytes via One-Step Sintering: Toward High Ionic Conductivity

Hao Zhang^1^, Yaocong Wang^1^, Quande Che^1^, Jiaming Wu^1^, Yanzhu Zhang^1^, Dongxu Mao^1^, Xundao Liu^1^, Jiajie Li ^1,*^, Zhengmao Ye^2^, Dehua Dong^3, 4, *^

1. School of Materials Science and Engineering, University of Jinan, Jinan, 250022, P. R. China
2. School of Civil Engineering and Architecture, Hainan University, Haikou, 570228, P. R. China
3. School of Materials Science and Engineering, Hainan University, Haikou, 570228, P. R. China
4. Moganshan Institute ZJUT, Kangqian District, Deqing 313200, P. R. China

* To whom correspondence should be addressed. E-mail: [mse_lijj@ujn.edu.cn](mailto:mse_lijj@ujn.edu.cn); dongdh7@hainanu.edu.cn; Tel: (+86) 531-89736011. Fax: (+86) 531-89736011.

**Experimental Section**

The pristine LLZO (p-LLZO), Al-doped, N-doped, and Al-N co-doped LLZO solid electrolytes were synthesized via a one-step solid-state reaction. Starting materials included LiOH (98.0%, Macklin), La_2_O_3_ (99.9%, Macklin), ZrO_2_ (<100 nm, Macklin), Al_2_O_3_ (99.8%, Macklin), AlN (99.9%, Macklin), Li_3_N (99.9%, Macklin), and ZrN (99.5%, Macklin). For the p-LLZO, Al-doped, and Al-N co-doped samples, the precursor powders were blended with anhydrous ethanol and wet-milled for 10 h at 350 rpm in a planetary ball mill using PTFE jars and ZrO_2_ balls, followed by drying at 100 °C for 10 h. Notably, for the N-doped LLZO samples, the mixing and milling processes were performed exclusively via dry-milling inside an Ar-filled glovebox (H_2_O, O_2_ < 0.1 ppm) to avoid the decomposition or oxidation of the nitrogen sources. The obtained powders were uniaxially pressed at 300 MPa for 1 min into pellets (Ø16 mm). The p-LLZO, Al-doped, and Al-N co-doped pellets were heated under an air atmosphere, while the N-doped pellets were sintered under a flowing N_2_ atmosphere. The heating profile for all samples consisted of a ramp of 2 °C/min to 900 °C (held for 5 h), followed by an increase to 1180 °C within 80 min (held for 10 min), and a final dwell at 1150 °C for 6 h. After natural cooling, the pellets were dry-polished sequentially with SiC abrasive papers (240, 600, 1000, 2000, and 5000 grit) and stored in an Ar-filled glovebox to prevent atmospheric contamination.

**Conventional solid electrolyte preparation:** Conventional process was also employed to make LLZO-Al_x_ (CPLLZO-Al_x_) electrolyte membranes by using LiOH, La_2_O_3_, ZrO_2_, and Al_2_O_3_ as starting materials. The powders were blended with anhydrous ethanol and continuously wet-milled for 10 h at 350 rpm in a planetary ball mill using PTFE jars and ZrO_2_ balls, and then the mixture was dried at 100 °C for 10 h. After drying, the mixture was calcined at 900 ℃ for 6 h at a heating rate of 5 ℃/min and then naturally cooled. The calcined powders were then subjected to a second round of ball milling to break agglomerations. Subsequently, the powders were uniaxially pressed at 300 MPa for 1 min into pellets (Ø16 mm), which were sintered in a muffle furnace (KSL-1700L) in air at 1200 °C for 5 h. These pellets were covered with mother powders to prevent Li loss during the high-temperature sintering process. All pellets were subjected to the same treatment as described above to ensure consistent surface conditions.

**Materials characterization**

The phase and crystal structures of the as-prepared pellets (p-LLZO, Al-doped, N-doped, and Al-N co-doped LLZO) were characterized by X-ray powder diffraction (XRD, Rigaku SmartLab 9 kW, Japan) in the 2θ range from 10°-80°. The elemental compositions and stoichiometric ratios of the samples were quantified using an inductively coupled plasma optical emission spectrometer (ICP-OES, iCAP PRO, Thermo Fisher Scientific, USA). Solid-state nuclear magnetic resonance (SSNMR) spectra were recorded on a 400 MHz spectrometer (Bruker, Germany). The microstructure of the sintered pellets was observed by a scanning electron microscope (Phenom Scientific, Holland) with energy dispersive spectrometer (EDS). The microstructural analysis of the LLZO-Al_0.50_N_0.50_ was performed using a JEM-2100F TEM (JEOL, Japan), and elemental distribution was recorded through energy-dispersive X-ray (EDX) spectroscopy mapping. X-ray photoelectron spectroscopy (XPS) characterizations were performed on an ESCALAB Xi+ instrument (Thermo Fisher Scientific, USA) and a K-Alpha spectrometer (Thermo Scientific, USA) to analyze the surface chemical states. Thermal properties were measured from 30 to 1200 ℃ at a heating rate of 10 ℃ min^-1^ in the air using thermogravimetric-differential scanning calorimetry (TG-DSC, PerkinElmer STA 8000, USA). The Archimedes method was used to measure the relative densities of sintered pellets, using ethanol as the immersion medium.

**Electrochemical measurements**

To measure the ionic conductivity, silver paste was applied on both sides of the pellets as blocking electrodes, followed by vacuum drying at 120 °C for 10h. The ionic conductivity of electrolyte membranes was measured via the AC impedance technique (EIS, CHI660E, China) in a frequency range from 1 MHz to 0.1 Hz with an amplitude of 0.01 V. For the electrochemical tests, Li|LLZO-Al_y_N_y_|Li symmetric cells were fabricated using the molten lithium method to obtain an intimate interface at 220 °C, and then assembled into coin cells for subsequent tests. Galvanostatic charge/discharge tests for the symmetric cells were performed at 25 °C on a LAND CT2001A test system. The critical current density (CCD) of the symmetric cells was measured using a stepwise current-increasing method at 25 °C.

In the process of fabricating the cathode electrode, a mixture was prepared by blending LFP, PVDF-HFP, and carbon nanotubes (CNTs) as a dispersant at a mass ratio of 8:1:1, followed by stirring for 6 h in NMP to obtain a uniformly dispersed slurry. The mixed slurry was coated onto aluminum (Al) foil and dried at 100℃ for 24 h. Then, the cathode film was cut into disks with a diameter of 10 mm to obtain LFP electrodes. The active material loading was about 2 mg cm^-2^. The LFP cathodes, LLZO-Al_y_N_y_ pellet and Li metal were assembled into 2032-coin cell in an Ar-filled glovebox with O^2^ and H_2_O concentrations lower than 0.1 ppm. 10 µL of 1.0 M LiTFSI in 1,3-Dioxolane and 1,2-Dimethoxyethane (v/v=1:1) solution was dropped between the LFP cathode and the solid electrolyte to improve the interfacial contact. The LFP full cells were galvanostatically charged/discharged in a potential range of 2.5-3.65 V at different current rates at 25℃. All cells were assembled in an argon-filled glovebox. Particularly, all cells were tested at 25°C on a LAND CT2001A battery test system.

**DFT calculations**

The DFT calculations were performed using the Vienna ab initio simulation package (VASP)^[1]^ with the Perdew–Burke–Ernzerhof (PBE) form of generalized gradient approximation (GGA)^[2]^ and projector-augmented wave (PAW) method^[3]^. Valence electrons considered for pure LLZO included 1s²2s¹ for Li, 5s²6s²5p⁶5d¹ for La, 4s²5s¹4p⁶4d³ for Zr, 2s²2p⁴ for O, 3s²3p¹ for Al and 2s^2^2p^3^ for N. To precisely represent the impact of N atom doping on the electronic structure, particularly the interaction of N's 2p electrons, the Hubbard correction^[4]^ was integrated into the GGA calculations (GGA+U) for LLZO-Al_y_N_y_. We did not apply the Hubbard correction for Zr and La as it has been suggested that it may be unnecessary for early-transition metals.^[5]^

During geometry optimization for p-LLZO, LLZO-Al_x_ and LLZO-Al_y_N_y_, we employed a plane-wave cutoff energy of **520 eV** and set the electronic convergence threshold to **1×10⁻⁶ eV** to ensure high precision in total energy calculations. The partial occupancies of electronic states were treated using the Gaussian smearing method with a smearing width of **0.05 eV**. All calculations utilized the **Gamma-centered k-point scheme** to sample the Brillouin zone. For structural relaxation, a **2×2×2 Gamma-only k-point mesh** was used to balance computational efficiency and accuracy, while a denser **3×3×3 Gamma mesh** was employed for partial density of states (PDOS) calculations to capture fine electronic structure details. The ionic relaxation convergence criterion was set to **-0.02 eV/Å**, corresponding to a maximum Hellmann-Feynman force of **0.02 eV Å⁻¹** on each atom.

To quantify the activation energy barrier of lithium-ion migration, the Nudged Elastic Band (NEB) method^[6]^ implemented in VASP was employed. The migration pathway was modeled as a ​96h → 24d → 96h hopping sequence, following the mechanism reported^[7]^. The initial and final states were optimized, and 5 intermediate images were inserted to map the minimum energy path (MEP).

The atomic structures of cubic Li₇La₃Zr₂O₁₂ (c-LLZO) were generated by enforcing two electrostatic rules governing Li⁺ distribution: (1) adjacent octahedral sites cannot be simultaneously occupied due to Coulomb repulsion, and (2) occupation of a tetrahedral site requires all neighboring octahedral sites to be vacant.^[8]^ To efficiently satisfy these constraints, ​40 Li ions were fixed in octahedral sites with maximized interatomic distances, while the remaining 16 Li ions were distributed as 11 in tetrahedral 24d sites and 5 in octahedral 96h sites, achieving the stable 60% tetrahedral/40% octahedral occupancy ratio.^[9]^ For doped systems (LLZO-Al_x_ and LLZO-Al_y_N_y_), Al³⁺ substitution at Li⁺ sites (with charge-balanced Li removal) or Al³⁺/N³⁻ co-substitution at Li⁺/O²⁻ sites was applied to the c-LLZO structure. All configurations were randomized via the Supercell program^[10]^, with 30 distinct samples generated for each system followed by DFT energy minimization to identify the lowest-energy structure.


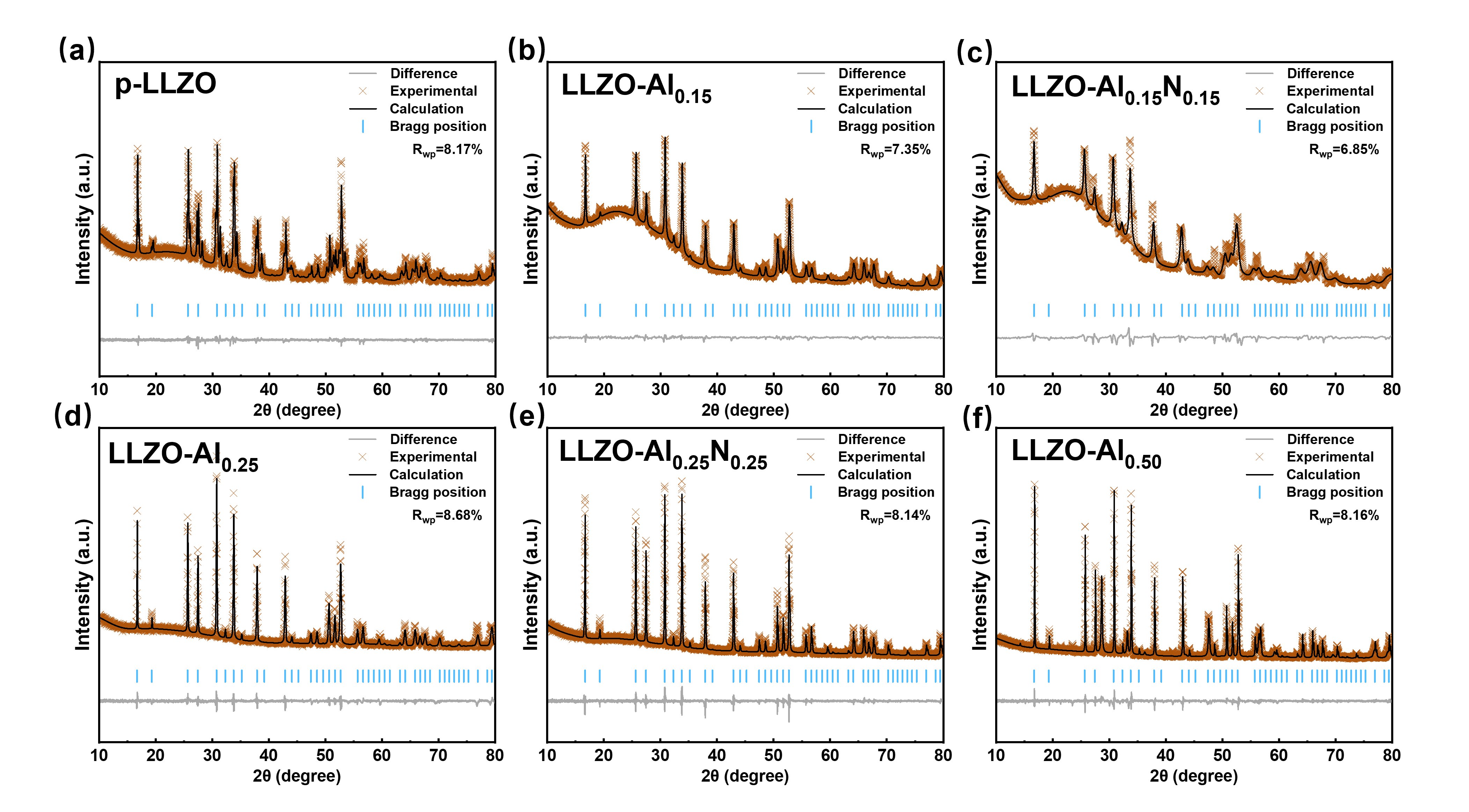


**Figure S1.** Rietveld refinement of XRD patterns for various LLZO samples. (a) p-LLZO, (b) LLZO-Al_0.15_, (c) LLZO-Al_015_N_0.15_, (d) LLZO-Al_0.25_, (e) LLZO-Al_0.25_N_0.25_, and (f) LLZO-Al_0.50_.


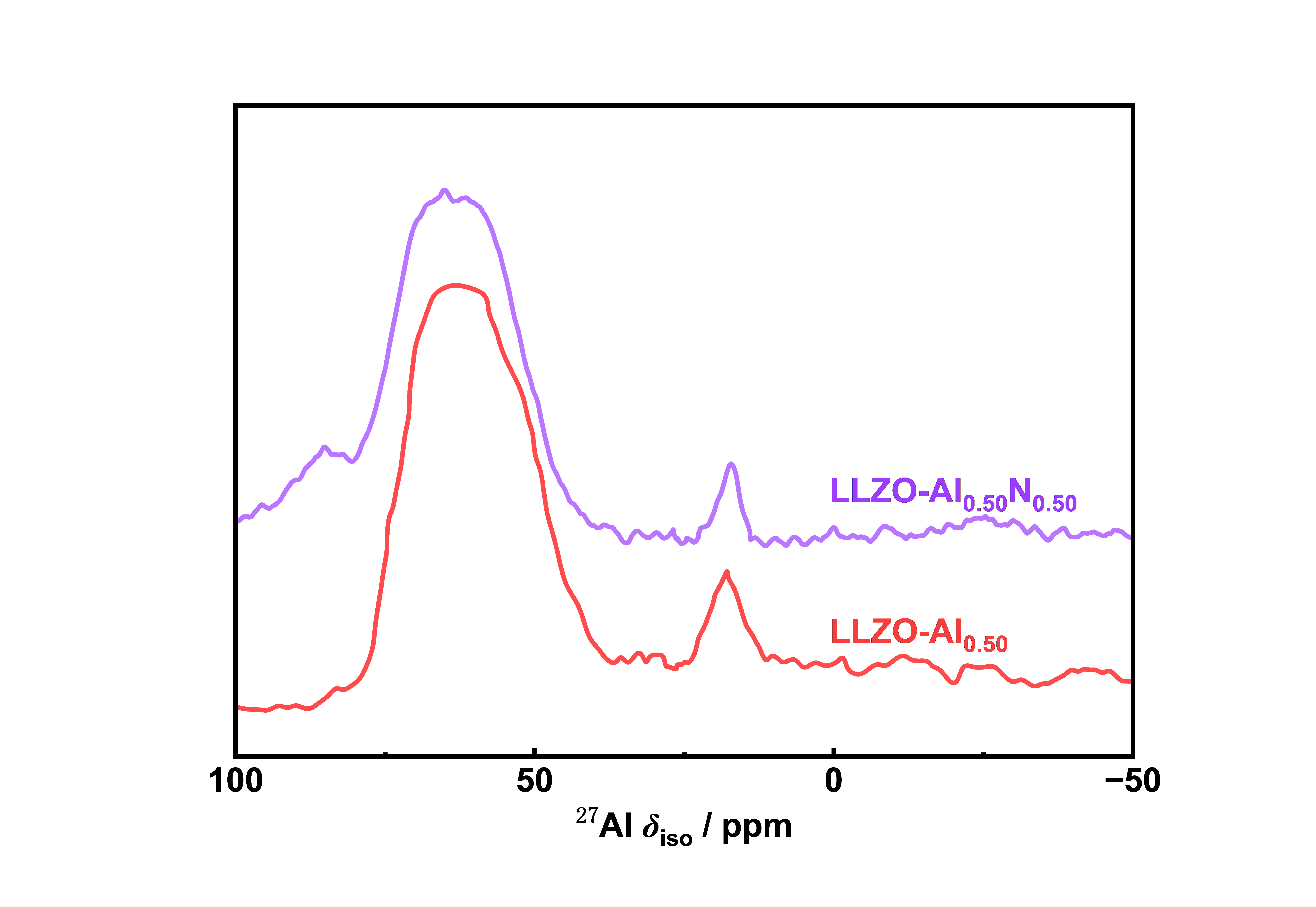


**Figure S2.** ^27^Al NMR spectra of LLZO-Al_0.50_ and LLZO-Al_0.50_N_0.50_.


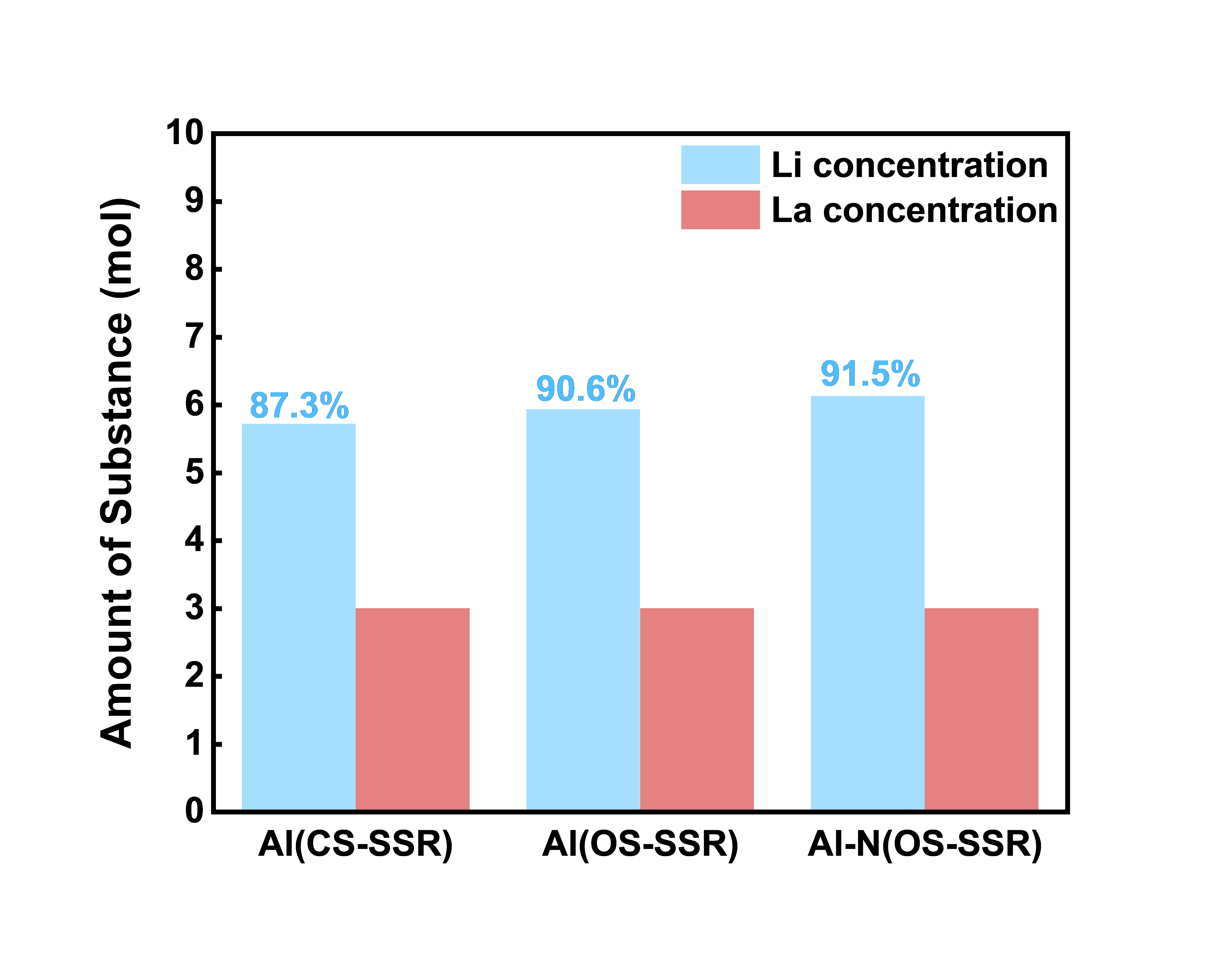


**Figure S3.** Elemental concentrations of Li and La for LLZO-Al_0.15_ (CS/OS-SSR) and LLZO-Al_0.15_N_0.15_ (OS-SSR) samples (Percentage represents the lithium retention rate).


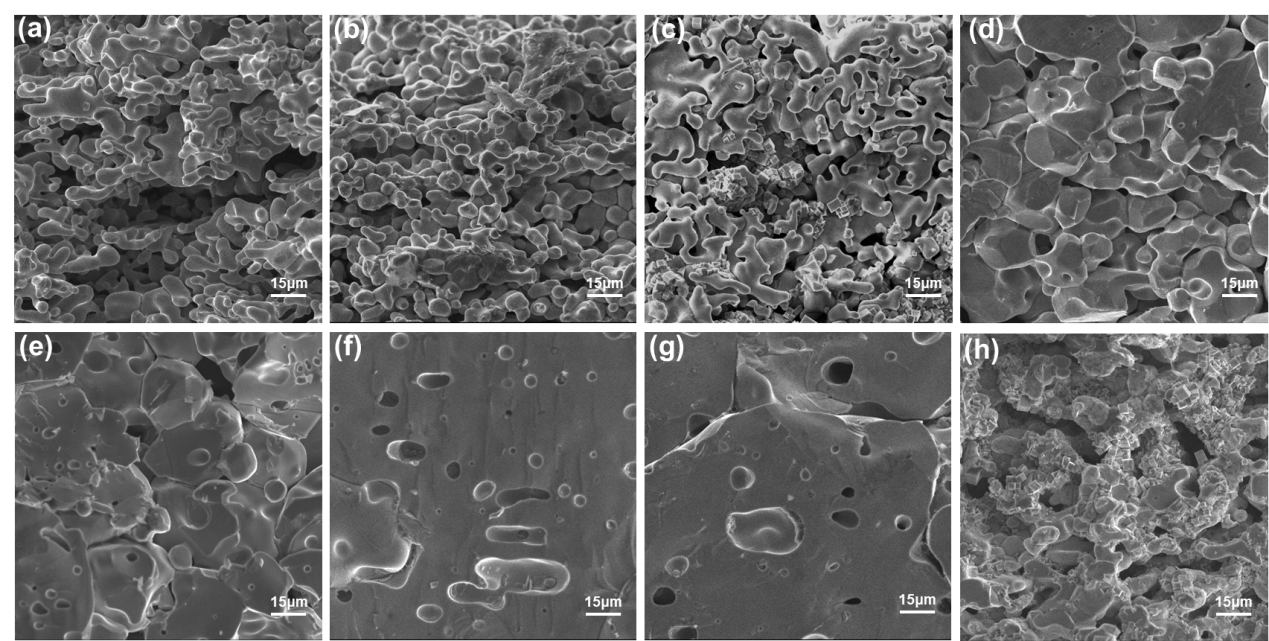


**Figure S4.** Cross-sectional SEM of sintered pellets of (a) LLZO, (b) LLZO-Al_0.15_, (c) LLZO-Al_0.50_, (d) LLZO-Al_0.15_N_0.15_, (e) LLZO-Al_0.25_N_0.25_, (f) LLZO-Al_0.50_N_0.50_, and (g) LLZO-Al_0.85_N_0.85_, (h) LLZO-Al_1.15_N_1.15_.


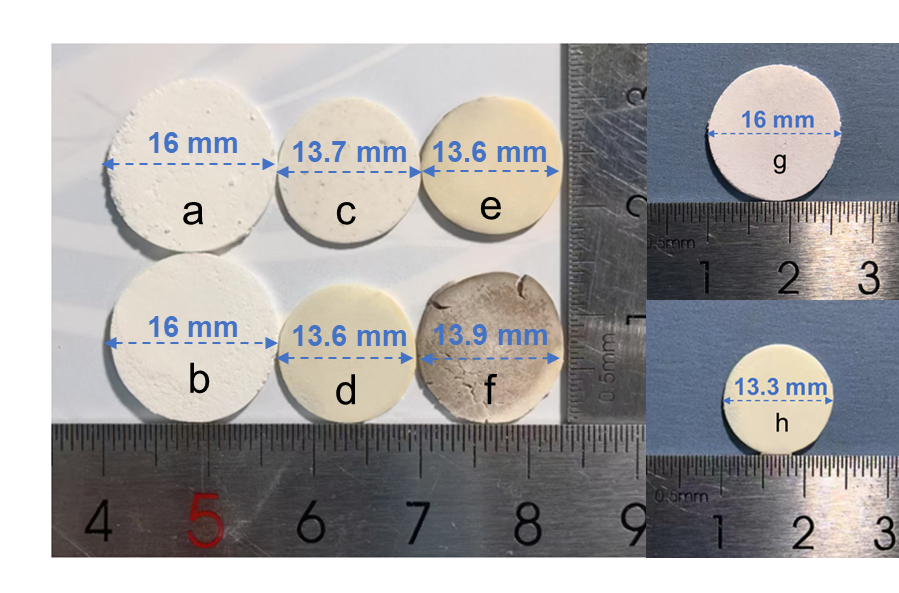


**Figure S5.** Digital photos the sintered LLZO pellets. (a) LLZO, (b) LLZO-Al_0.15_, (c) LLZO-Al_0.15_N_0.15_, (d) LLZO-Al_0.25_N_0.25_, (e) LLZO-Al_0.85_N_0.85_, (f) LLZO-Al_1.15_N_1.15_, (g) LLZO-Al_0.50_, and (h) LLZO-Al_0.50_N_0.50_.


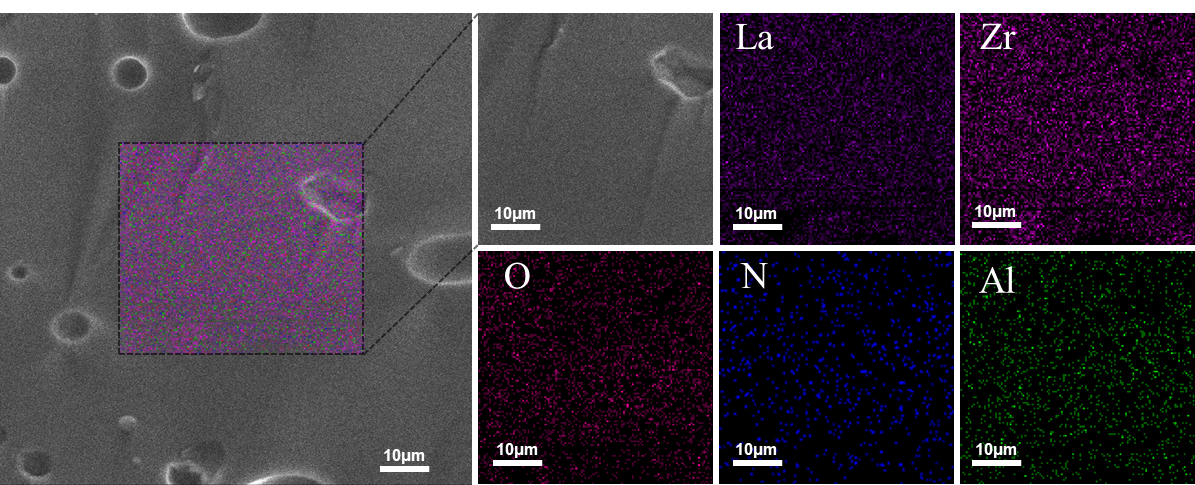


**Figure S6.** Cross-sectional SEM image and corresponding EDX elemental maps of LLZO-Al_0.50_N_0.50_.


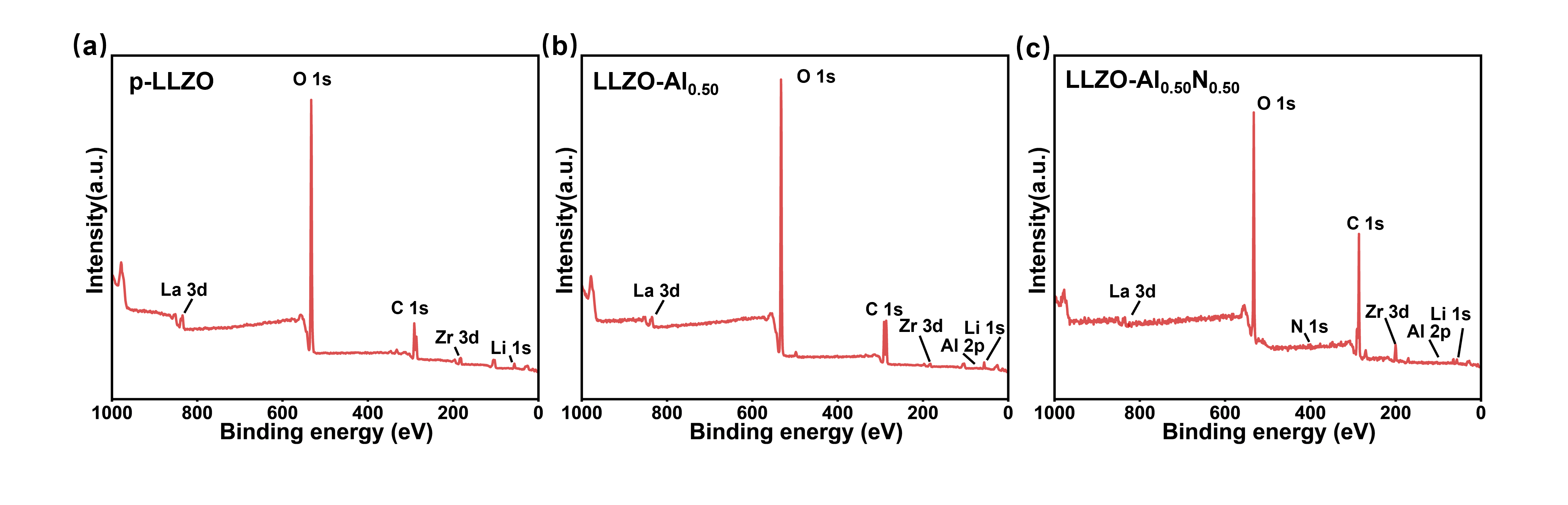


**Figure S7.** XPS survey spectra of the LLZO samples. (a) LLZO, (b) LLZO-Al_0.50_, and (c) LLZO-Al_0.50_N_0.50_.


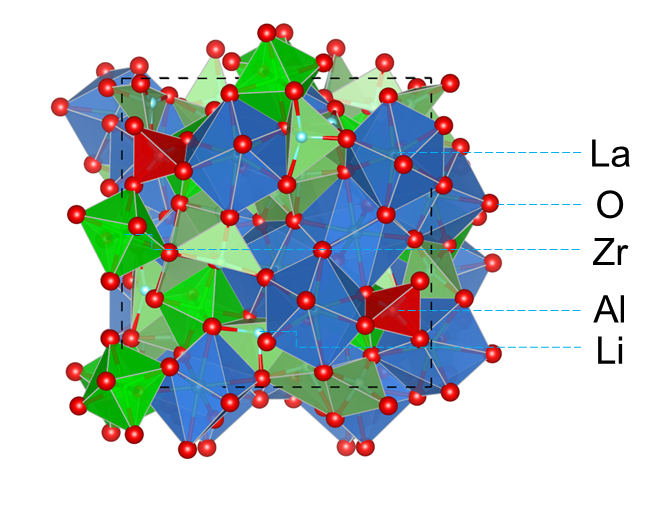


**Figure S8.** Schematic crystal structure of LLZO-Al_0.50_.


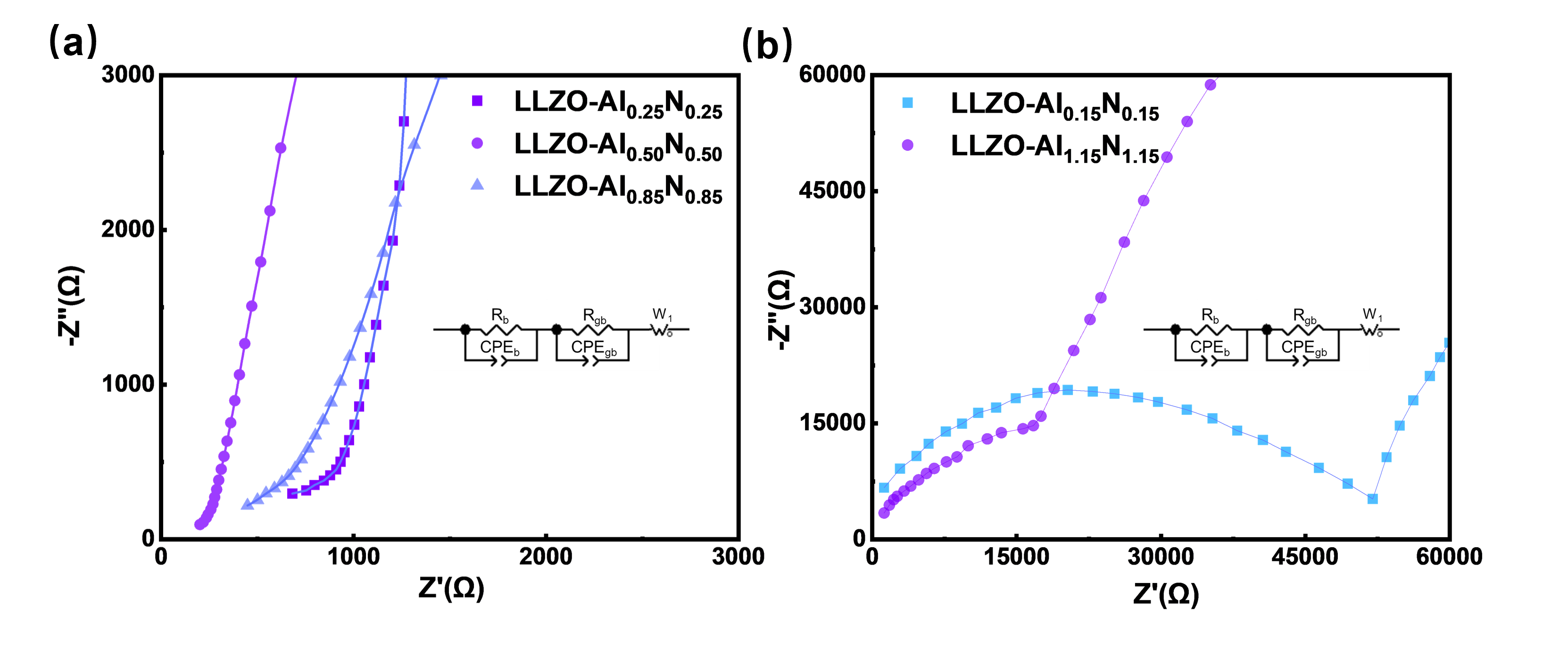


**Figure S9.** EIS Nyquist plots of LLZO-Al_y_N_y_ samples. (a) Nyquist plots for y = 0.25, 0.50, and 0.85. (b) Nyquist plots for y = 0.15 and 1.15.


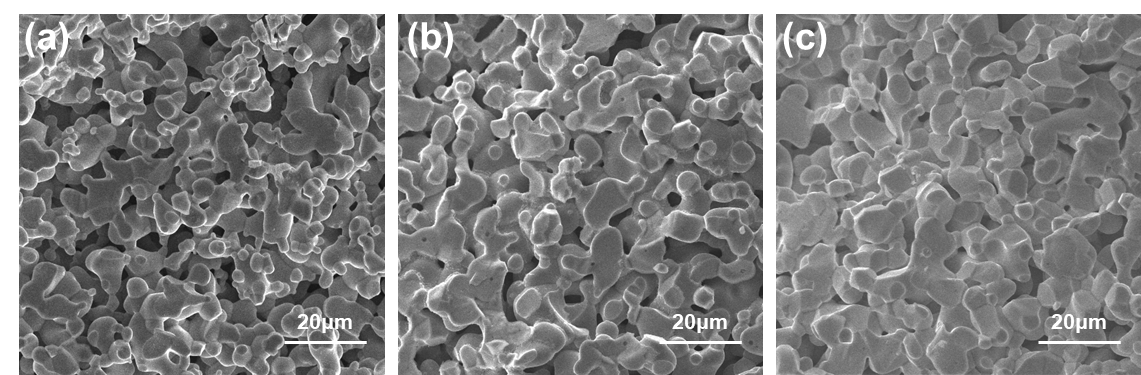


**Figure S10.** Cross-sectional SEM of sintered pellets of (a) CPLLZO-Al_0.15_, (b) CPLLZO-Al_0.25_, and (c) CPLLZO-Al_0.50_.


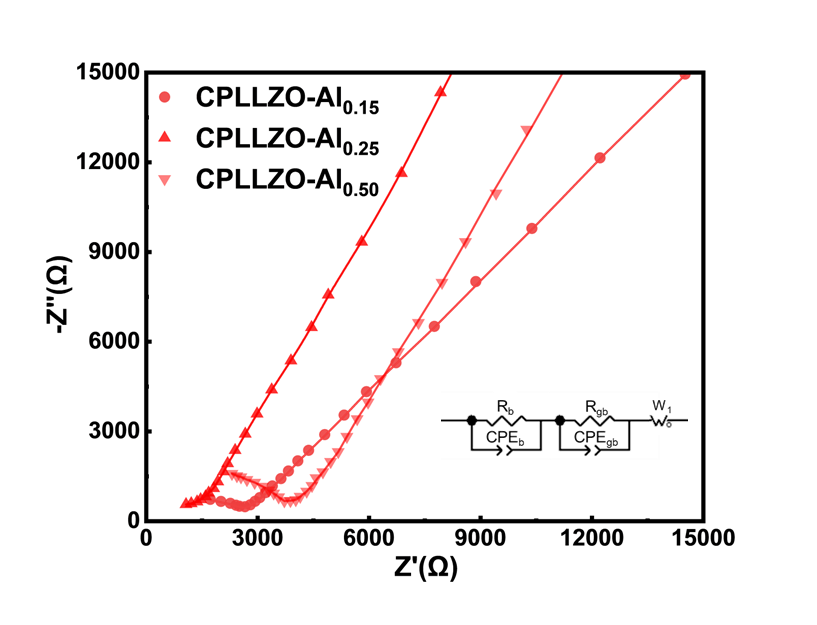


**Figure S11.** EIS Nyquist plots of CPLLZO-Al_x_.

**
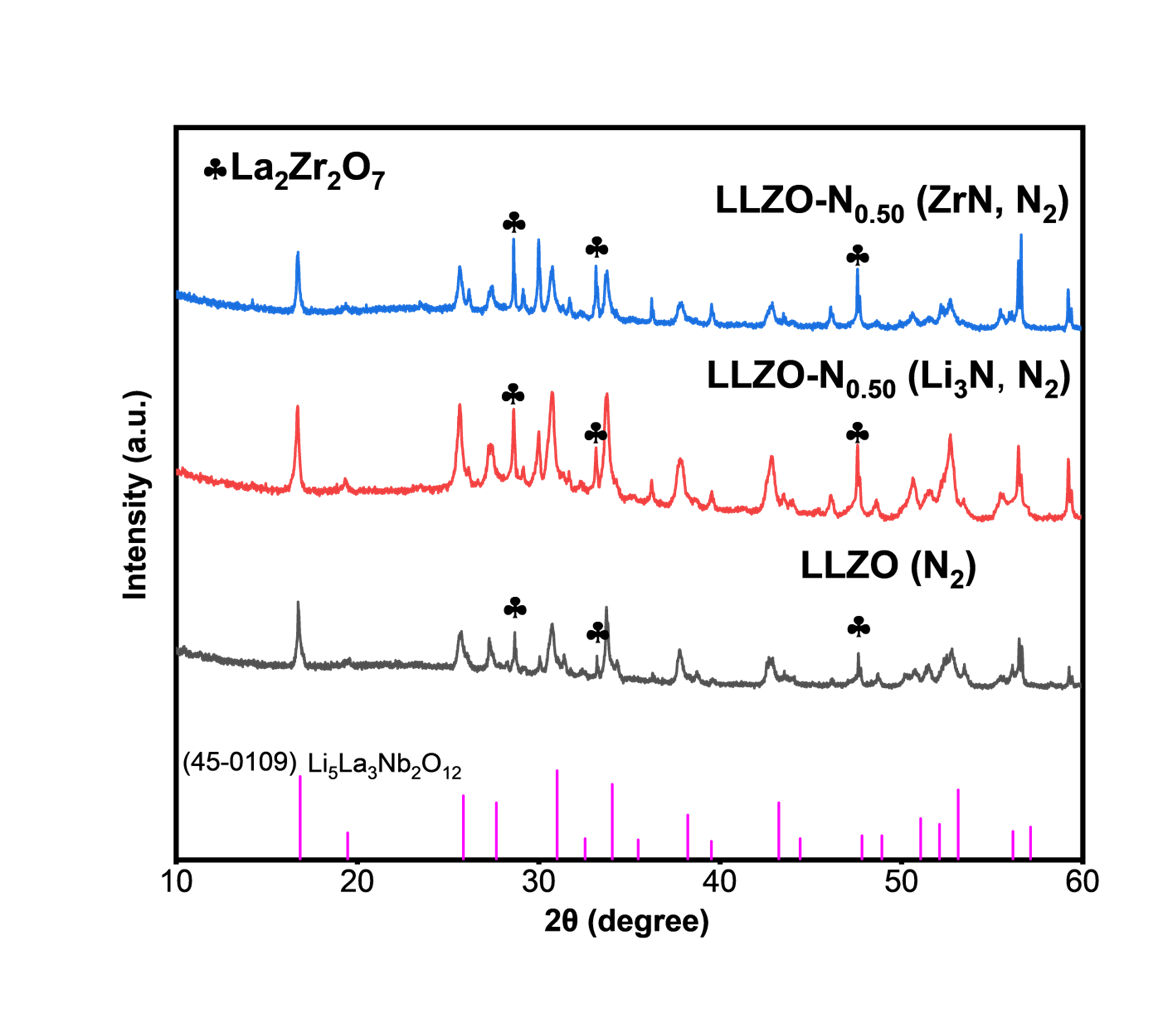
**

**Figure S12.** XRD patterns of LLZO (N_2_), LLZO-N_0.50_ (Li_3_N, N_2_), and LLZO-N_0.50_ (ZrN, N_2_).


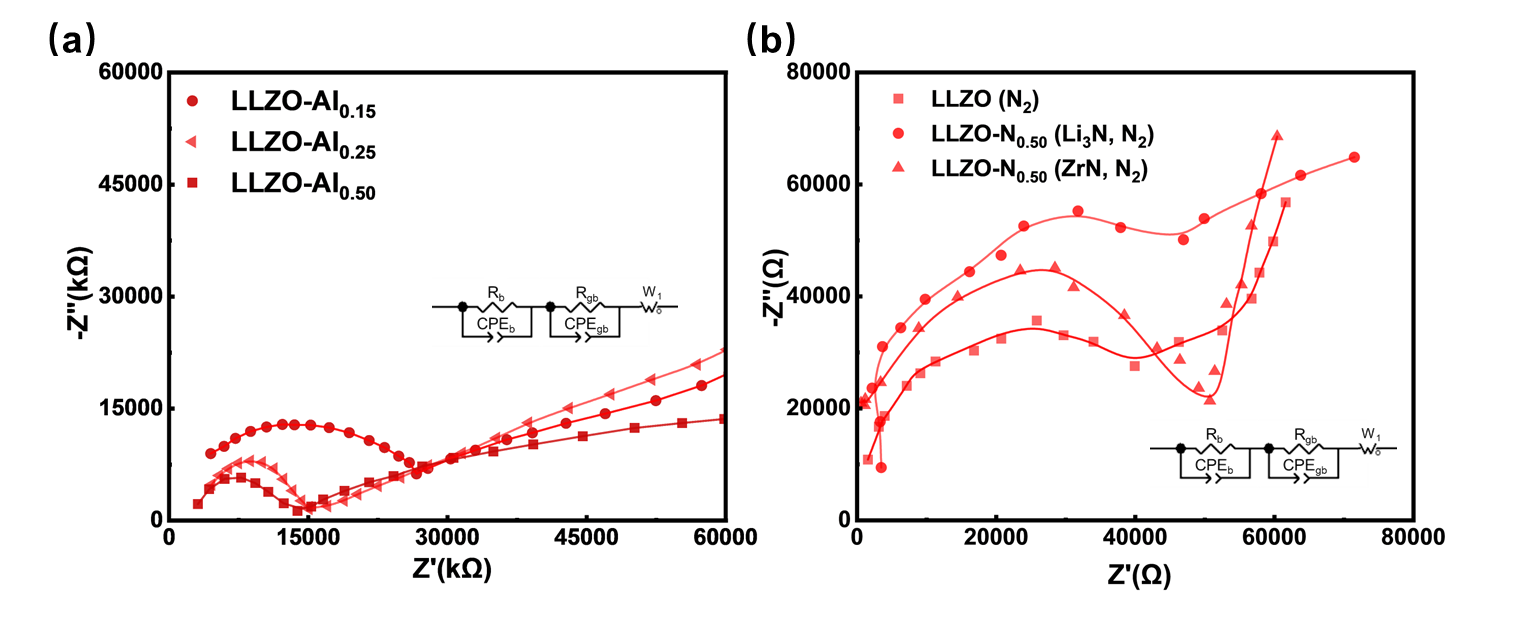


**Figure S13.** EIS Nyquist plots of (a) Al-doped LLZO and (b) N-doped LLZO.


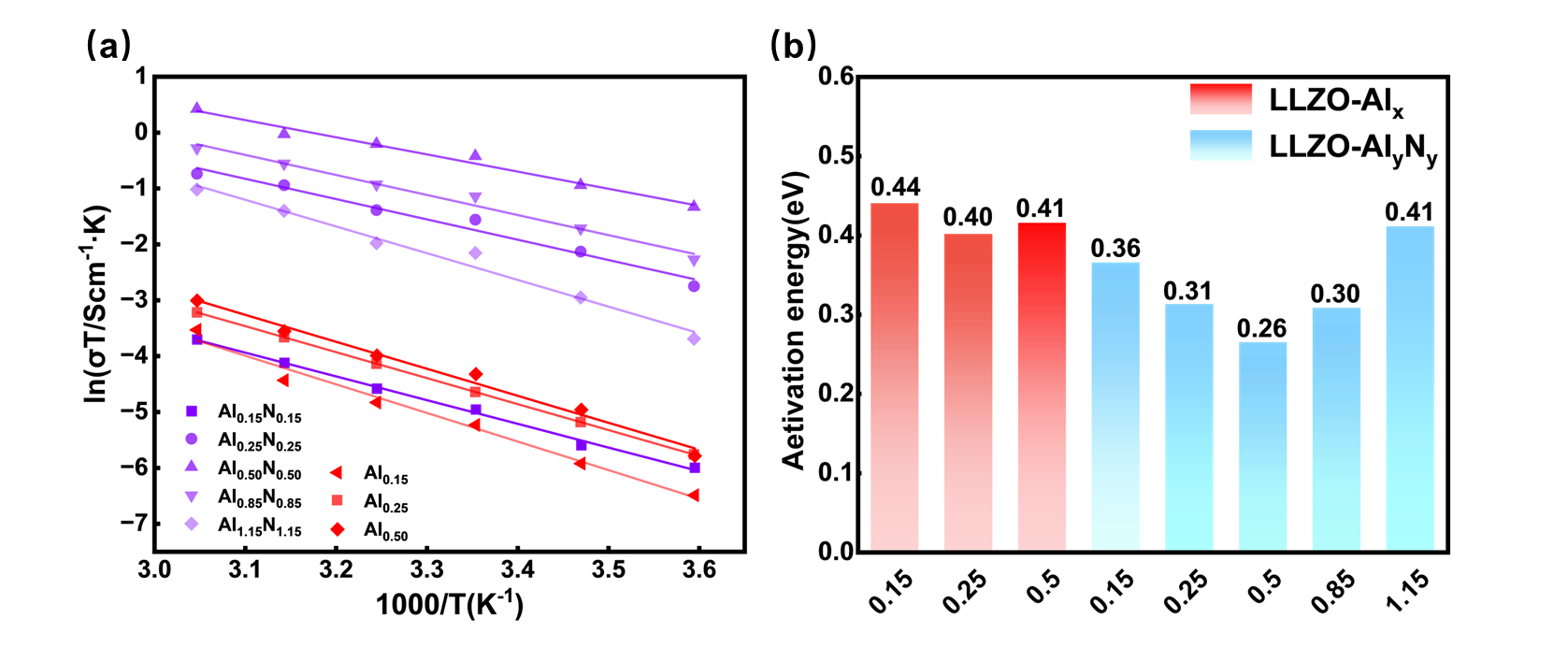


**Figure S14.** (a) Arrhenius plots of LLZO-Al_x_ and LLZO-Al_y_N_y_. (b) Activation energy of LLZO-Al_x_ and LLZO-Al_y_N_y_.


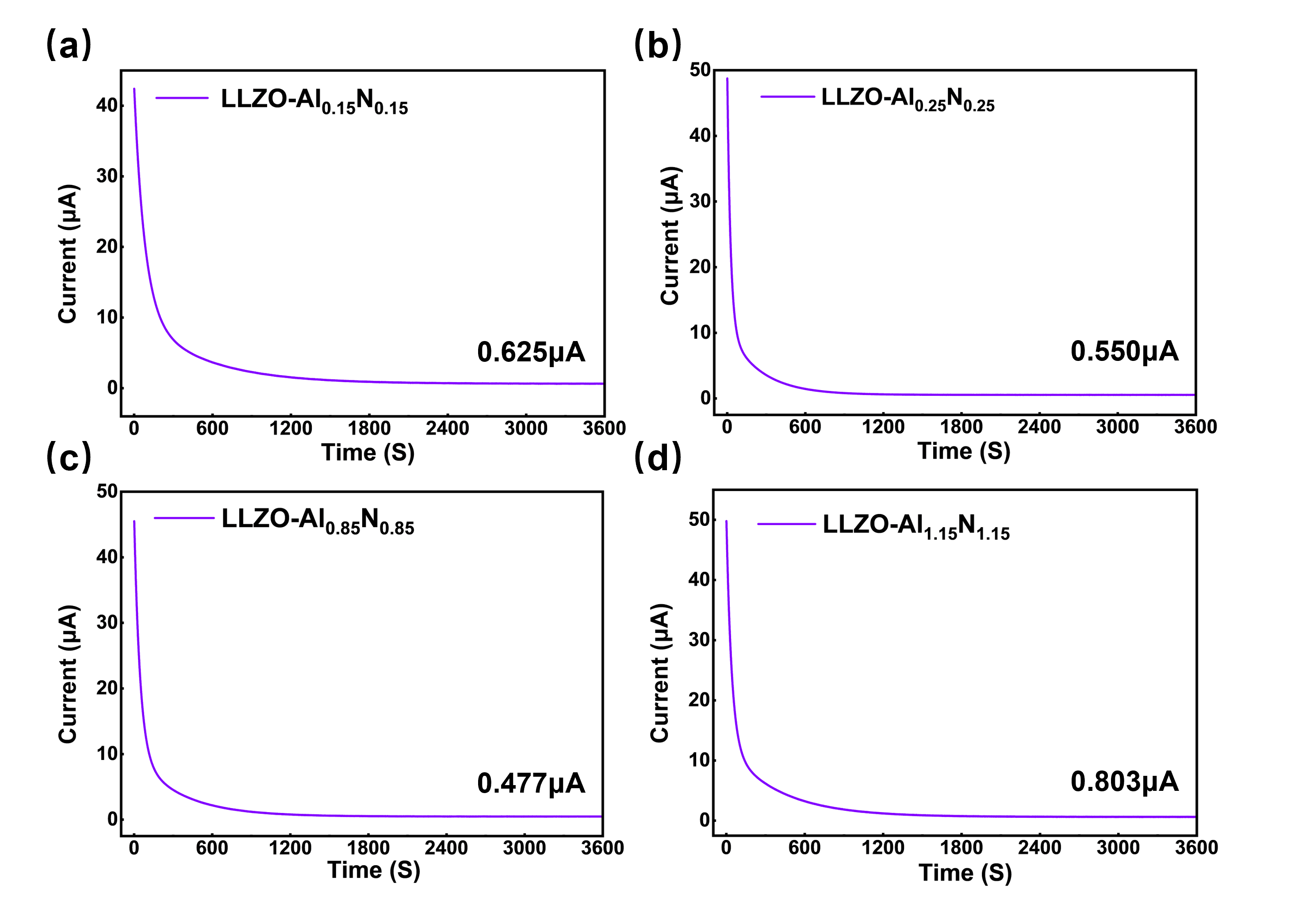


**Figure S15.** DC polarization curves of LLZO-Al_y_N_y_ samples. (a) y = 0.15, (b) y = 0.25, (c) y = 0.85, and (d) y = 1.15.


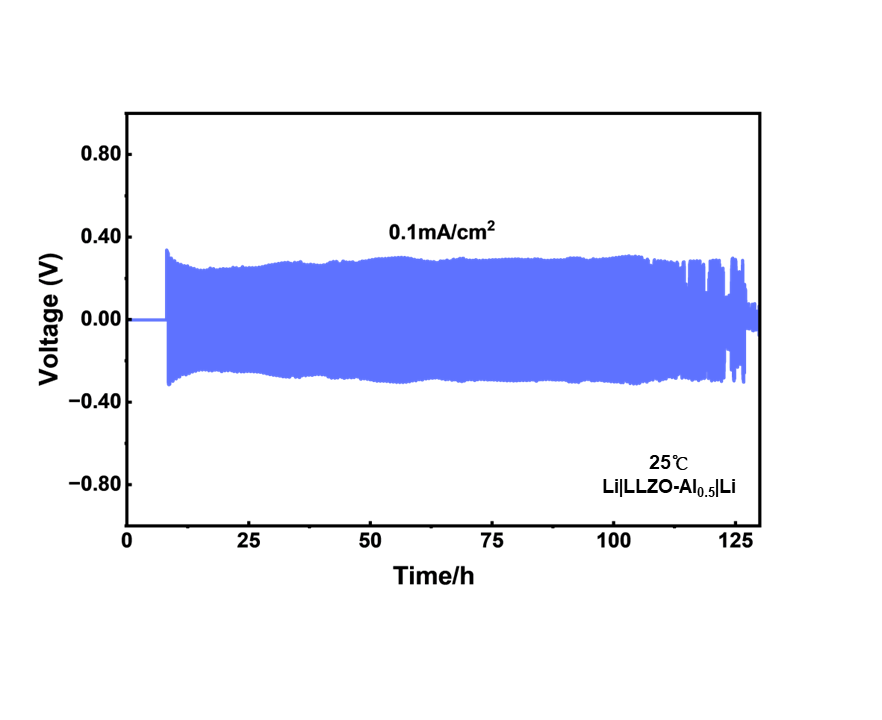


**Figure S16.** Galvanostatic cycling test of the symmetric Li|LLZO-Al_0.5_|Li cell at 0.1 mA cm^−2^.


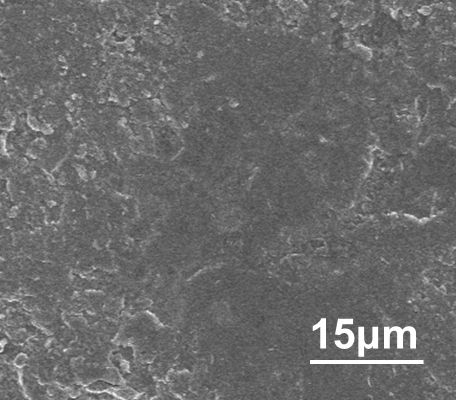


**Figure S17.** Surface SEM image of the LLZO-Al_0.50_N_0.50_ electrolyte after symmetric cell cycling.


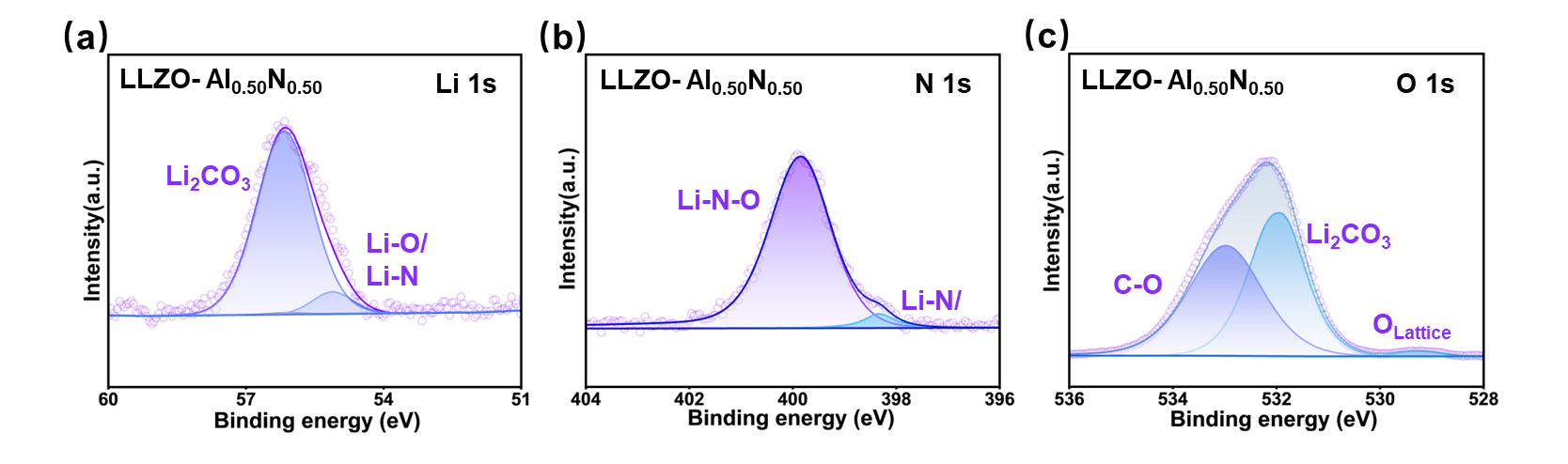


**Figure S18.** High-resolution XPS spectra of the LLZO-Al_0.50_N_0.50_ electrolyte surface after symmetric cell cycling. (a) Li 1s, (b) N 1s, and (c) O 1s spectra.


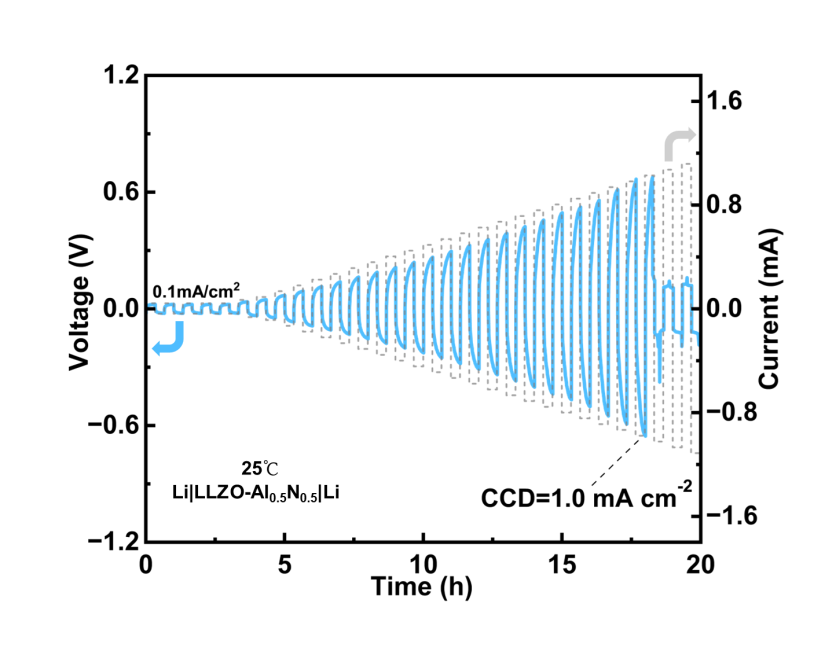


**Figure S19.** Potential responses of the symmetric Li|LLZO-Al_0.50_N_0.50_|Li cell during the critical current density measurement using time-constant mode at 25 ℃.

**
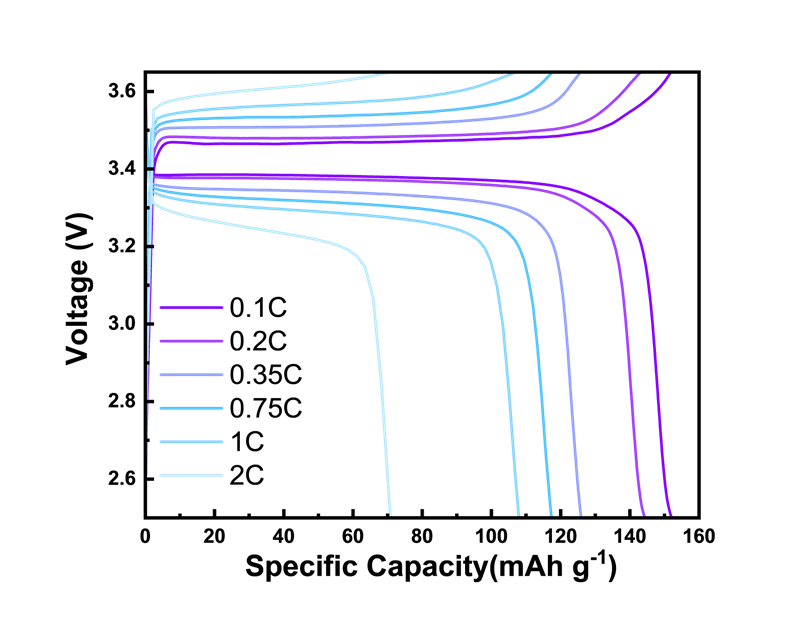
**

**Figure S20.** (a) Charge/discharge curves of the LFP|LLZO-Al_0.50_N_0.50_|Li batteries with the cathode loading of ≈ 2.0 mg cm^−2^ for cycles at 0.1, 0.2, 0.35, 0.75, 1, and 2C, respectively.

**Table S1.** Structural parameters of the electrolytes.

| Sample | Structure type and purity (%) | Anion N Occupancy (Occ_N_) | Lattice parameter (Å) |
| --- | --- | --- | --- |
| Li_7_La_3_Zr_2_O_12_ | Tetragonal (72.7) + cubic (27.3) | - | *a=b=*13.1128, *c=*12.7004 |
| Li_6.55_Al_0.15_La_3_Zr_2_O_12_ | Tetragonal (26.8) + cubic (72.2) | - | 12.9829 |
| Li_6.70_Al_0.15_La_3_Zr_2_O_11.85_N_0.15_ | Tetragonal (47.5) + cubic (52.5) | 0.0097 | 13.0371 |
| Li_6.25_Al_0.25_La_3_Zr_2_O_12_ | cubic (Phase-pure^a^) | - | 12.9601 |
| Li_6.50_Al_0.25_La_3_Zr_2_O_11.75_N_0.25_ | cubic (Phase-pure) | 0.0176 | 12.9765 |
| Li_5.50_Al_0.50_La_3_Zr_2_O_12_ | cubic (~90%) | - | 12.9537 |
| Li_6.00_Al_0.50_La_3_Zr_2_O_11.50_N_0.50_ | cubic (Phase-pure) | 0.0403 | 12.9650 |

^a^ No secondary phases were detected within the detection limits of the XRD instrument.

**Table S2.** Lithium Site Occupancy and Vacancy Distribution Derived from ^7^Li ss-NMR.

| Sample | Site | Area Fraction (%) | Occupancy (per p.f.u.) | Vacancy (per p.f.u.) |
| --- | --- | --- | --- | --- |
| LLZO-Al_0.50_ | Li_1_ (24d) | 26.8 | 1.43 | 1.57 |
|  | Li_2_ (96h) | 73.2 | 4.07 | 7.93 |
| LLZO-Al_0.50_N_0.50_ | Li_1_ (24d) | 36.4 | 2.16 | 0.84 |
|  | Li_2_ (96h) | 63.6 | 3.84 | 8.16 |

**Table S3.** Summary of XPS analysis results for p-LLZO.

| Core Level | Binding Energy (eV) | FWHM (eV) | Peak Assignment | Relative Area (%) |
| --- | --- | --- | --- | --- |
| C 1s | 284.80 | 1.22 | C-C / C-H | 55.1 |
|  | 289.84 | 1.16 | O-C=O | 44.9 |
| Li 1s | 54.44 | 1.13 | Li-O | 40.3 |
|  | 55.05 | 1.03 | Li_2_CO_3_ | 59.7 |
| Al 2p | - | - | - | - |
| N 1s | - | - | - | - |
| O | 528.77 | 1.36 | O_Lattice_ | 7.9 |
|  | 531.62 | 1.26 | Li_2_CO_3_ | 72.3 |
|  | 532.21 | 1.17 | C-O | 19.8 |

**Table S4.** Summary of XPS analysis results for LLZO-Al_0.50_.

| Core Level | Binding Energy (eV) | FWHM (eV) | Peak Assignment | Relative Area (%) |
| --- | --- | --- | --- | --- |
| C 1s | 284.82 | 1.19 | C-C / C-H | 54.7 |
|  | 289.93 | 1.17 | O-C=O | 45.3 |
| Li 1s | 54.72 | 1.09 | Li-O | 41.3 |
|  | 55.22 | 1.25 | Li_2_CO_3_ | 58.7 |
| Al 2p | 73.13 | 1.58 | Al-O | 100.0 |
| N 1s | - | - | - | - |
| O | 530.01 | 1.96 | O_Lattice_ | 5.4 |
|  | 531.70 | 1.29 | Li_2_CO_3_ | 62.7 |
|  | 532.28 | 1.48 | C-O | 31.9 |

**Table S5.** Summary of XPS analysis results for LLZO-Al_0.50_N_0.50_.

| Core Level | Binding Energy (eV) | FWHM (eV) | Peak Assignment | Relative Area (%) |
| --- | --- | --- | --- | --- |
| C 1s | 284.82 | 1.19 | C-C / C-H | 54.7 |
|  | 289.93 | 1.16 | O-C=O | 45.3 |
| Li 1s | 54.52 | 1.38 | Li-O / Li-N | 39.7 |
|  | 55.22 | 1.23 | Li_2_CO_3_ | 60.3 |
| Al 2p | 72.71 | 1.59 | Al-O | 100.0 |
| N 1s | 399.47 | 1.57 | Li-N-O | 100.0 |
| O | 529.14 | 1.00 | O_Lattice_ | 5.2 |
|  | 531.58 | 1.65 | Li_2_CO_3_ | 82.0 |
|  | 532.77 | 1.44 | C-O | 12.8 |

**Table S6.** Summary of physical properties and electrochemical performance of the samples.

| Composition | R_b_ (Ω) | R_gb_ (Ω) | 𝜎Li^+^ [S cm^−1^]^a^ | Rd [%] ^b^ | Method |
| --- | --- | --- | --- | --- | --- |
| Li_7_La_3_Zr_2_O_12_ | - | - | - | 64.3 | OS-SSR^c^ |
| Li_6.55_Al_0.15_La_3_Zr_2_O_12_ | 4481 | 10728 | 3.30 × 10^-5^ | 70.2 | OS-SSR |
| Li_6.25_Al_0.25_La_3_Zr_2_O_12_ | 4587 | 10483 | 3.43 × 10^-5^ | 77.3 | OS-SSR |
| Li_5.50_Al_0.50_La_3_Zr_2_O_12_ | 3112 | 22199 | 2.01 × 10^-5^ | 75.7 | OS-SSR |
| Li_7_La_3_Zr_2_O_12_ (N_2_) | 1583 | 38322 | 1.34 × 10^-5^ | 82.3 | OS-SSR |
| Li_7.5_La_3_Zr_2_O_11.5_N_0.5_ (Li_3_N, N_2_) | 3504 | 43506 | 1.17 × 10^-5^ | 83.4 | OS-SSR |
| Li_7.5_La_3_Zr_2_O_11.5_N_0.5_ (ZrN, N_2_) | 1200 | 49610 | 1.02 × 10^-5^ | 80.2 | OS-SSR |
| Li_6.70_Al_0.15_La_3_Zr_2_O_11.85_N_0.15_ | 1237 | 15513 | 2.85 × 10^-5^ | 86.2 | OS-SSR |
| Li_6.50_Al_0.25_La_3_Zr_2_O_11.75_N_0.25_ | 450 | 214 | 0.72 × 10^-3^ | 92.9 | OS-SSR |
| Li_6.00_Al_0.50_La_3_Zr_2_O_11.50_N_0.50_ | 201 | 58 | 2.19 × 10^-3^ | 95.3 | OS-SSR |
| Li_5.30_Al_0.85_La_3_Zr_2_O_11.15_N_0.85_ | 681 | 228 | 0.96 × 10^-3^ | 93.6 | OS-SSR |
| Li_4.70_Al_1.15_La_3_Zr_2_O_10.85_N_1.15_ | 1231 | 50769 | 1.09 × 10^-5^ | 82.7 | OS-SSR |
| Li_6.55_Al_0.15_La_3_Zr_2_O_12_ | 1730 | 934 | 1.94 × 10^-4^ | 91.7 | CP^d^ |
| Li_6.25_Al_0.25_La_3_Zr_2_O_12_ | 1078 | 744 | 3.06 × 10^-4^ | 93.9 | CP |
| Li_5.50_Al_0.50_La_3_Zr_2_O_12_ | 2314 | 1578 | 1.36 × 10^-4^ | 93.4 | CP |

^a^ Li-ion conductivity at room temperature; ^b^ Relative density; ^c^ One-step solid-state reaction/sintering (OS-SSR); ^d^ Conventional preparation (CP).

**Table S7.** Summary of physical properties and electrochemical performance of the samples.

| Composition | Steady-state Current (μA) | 𝜎_e_ [S cm^−1^] |
| --- | --- | --- |
| Li_6.70_Al_0.15_La_3_Zr_2_O_11.85_N_0.15_ | 0.625 | 5.97 × 10^-7^ |
| Li_6.50_Al_0.25_La_3_Zr_2_O_11.75_N_0.25_ | 0.550 | 5.26 × 10^-7^ |
| Li_6.00_Al_0.50_La_3_Zr_2_O_11.50_N_0.50_ | 0.409 | 4.64 × 10^-7^ |
| Li_5.30_Al_0.85_La_3_Zr_2_O_11.15_N_0.85_ | 0.477 | 5.16 × 10^-7^ |
| Li_4.70_Al_1.15_La_3_Zr_2_O_10.85_N_1.15_ | 0.803 | 9.07 × 10^-7^ |
| Li_5.50_Al_0.50_La_3_Zr_2_O_12_ | 0.307 | 3.25 × 10^-7^ |

**Table S8.** Substitution with heteroatoms of cubic LLZO and their properties.

| Composition | Dopant | 𝜎 [S cm^−1^] ^a^ | E_a_ (eV) | Rd [%] ^b^ | Method | ref | |
| --- | --- | --- | --- | --- | --- | --- | --- |
|  |  |  |  |  |  |  | |
| Li_6.4_La_3_Zr_1.4_Ta_0.6_O_12_ | Ta^5+^ | 4.19 × 10^−4^ | 0.43 | 93.9 | CSSR | ^[11]^ | |
| Li_6.4_La_3_Zr_1.4_Ta_0.6_O_12_ | Ta^5+^ | 6.3 × 10^-4^ | - | ≈95 | CSSR | ^[12]^ | |
| Li_6.55_La_3_Zr_2_Al_0.15_O_12_ | Al^3+^ | 4.4 × 10^−6^ | 0.49 | 51 | NSP | ^[13]^ | |
| Li_6.25_Al_0.25_La_3_Zr_2_O_12_ | Al^3+^ | 9.13 × 10^−6^ | - | - | MSS | ^[14]^ | |
| Li_6.25_Al_0.25_La_3_Zr_2_O_12_ | Al^3+^ | 5.89 × 10^−4^ | - | 94 | Sol–gel | ^[15]^ | |
| Li_6.25_Al_0.25_La_3_Zr_2_O_12_ | Al^3+^ | 1.8 × 10^−4^ | 0.25 | 89.83 | CSSR | ^[16]^ | |
| Li_6.4_Ga_0.2_La_3_Zr_2_O_12_ | Ga^3+^ | 1.24 × 10^−3^ | 0.31 | 97.3 | CSSR | ^[17]^ | |
| Li_6.25_Ga_0.25_La_3_Zr_2_O_12_ | Ga^3+^ | 1.30 × 10^−3^ | 0.28 | - | CSSR | ^[18]^ | |
| Li_6.25_Ga_0.25_La_3_Zr_2_O_12_ | Ga^3+^ | 1.46 × 10^-3^ | 0.25 | 94.1 | CSSR | ^[19]^ | |
| Li_6.4_Fe_0.2_La_3_Zr_2_O_12_ | Fe^3+^ | 4.28 × 10^-4^ | 0.27 | 95.6 | Pechini | ^[20]^ | |
| Li_7.2_La_3_Zr_1.8_Gd_0.2_O_12_ | Gd | 2.3 × 10^-4^ | 0.25 | 93-95 | CSSR | ^[21]^ | |
| Li_6.6_Mg_0.2_La_3_Zr_2_O_12_ | Mg^2+^ | 1.1 × 10^−4^ | - | 93 | Sol-gel | ^[22]^ | |
| Li_6.8_Al_0.2_La_3_Zr_2_O_11.8_F_0.2_ | Al^3+^、F^-^ | 7.45 × 10^−4^ | 0.288 | > 95.4 | CSSR | ^[23]^ | |
| Li_6.2_Al_0.2_La_3_Zr_2_O_11.8_F_0.2_ | Al^3+^、F^-^ | 3.18 × 10^−4^ | 0.24 | 91 | CSSR | ^[24]^ | |
| Li_6.05_Ga_0.25_La_3_Zr_2_O_11.8_F_0.2_ | Ga^3+^、F^-^ | 1.28 × 10^−3^ | 0.28 | - | CSSR | ^[25]^ | |
| Li_6.00_Al_0.5_La_3_Zr_2_O_11.50_N_0.5_ | Al^3+^、N^3-^ | 2.19 × 10^-3^ | 0.26 | 95.39 | OS-SSR | | ☆ |

^a^ Li-ion conductivity (room temperature); ^b^ Relative density; ☆: This work.

**Reference**

[1] G. Kresse, J. Furthmüller, *PHYS REV B* **1996**, *54*, 11169.

[2] J. P. Perdew, K. Burke, M. Ernzerhof, *Phys. Rev. Lett.* **1996**, *77*, 3865.

[3] G. Kresse, D. Joubert, *PHYS REV B* **1999**, *59*, 1758.

[4] J. Hubbard, *P Roy Soc A-math Phy* **1963**, *276*, 238-257.

[5] T. Thompson, S. Yu, L. Williams, R. D. Schmidt, R. Garcia-Mendez, J. Wolfenstine, J. L. Allen, E. Kioupakis, D. J. Siegel, J. Sakamoto, *ACS Energy Letters* **2017**, *2*, 462-468.

[6] G. Mills, H. Jónsson, G. K. Schenter, *Surface Science* **1995**, *324*, 305-337.

[7] C. Shi, J. Song, Y. Zhang, X. Wang, Z. Jiang, T. Sun, J. Zhao, *Cell Rep. Phys. Sci.* **2023**, *4*.

[8] H. Xie, J. A. Alonso, Y. Li, M. T. Fernandez-Diaz, J. B. Goodenough, *Chem. Mater.* **2011**, *23*, 3587-3589.

[9] A. Moradabadi, P. Kaghazchi, *Solid State Ionics* **2019**, *338*, 74-79.

[10] K. Okhotnikov, T. Charpentier, S. Cadars, *J. Cheminf.* **2016**, *8*, 17.

[11] X. Huang, Y. Lu, J. Jin, S. Gu, T. Xiu, Z. Song, M. E. Badding, Z. Wen, *ACS Appl. Mater. Interfaces* **2018**, *10*, 17147-17155.

[12] G. Lu, W. Liu, Z. Yang, Y. Wang, W. Zheng, R. Deng, R. Wang, L. Lu, C. Xu, *Adv. Funct. Mater.* **2023**, *33*, 2304407.

[13] R. Djenadic, M. Botros, C. Benel, O. Clemens, S. Indris, A. Choudhary, T. Bergfeldt, H. Hahn, *Solid State Ionics* **2014**, *263*, 49-56.

[14] Y. Zhang, A. Liu, Z. Shi, S. Ge, J. Zhang, *Int. J. Appl. Ceram. Technol.* **2022**, *19*, 320-331.

[15] M. Ashuri, M. Golmohammad, A. Soleimany Mehranjani, M. Faghihi Sani, *J MATER SCI-MATER EL* **2021**, *32*, 6369-6378.

[16] C. Chen, Y. Sun, L. He, M. Kotobuki, E. Hanc, Y. Chen, K. Zeng, L. Lu, *ACS Appl. Energy Mater.* **2020**, *3*, 4708-4719.

[17] J. Su, X. Huang, Z. Song, T. Xiu, M. E. Badding, J. Jin, Z. Wen, *Ceram. Int.* **2019**, *45*, 14991-14996.

[18] E. Yi, W. Wang, J. Kieffer, R. M. Laine, *J. Power Sources* **2017**, *352*, 156-164.

[19] J.-F. Wu, E.-Y. Chen, Y. Yu, L. Liu, Y. Wu, W. K. Pang, V. K. Peterson, X. Guo, *ACS Appl. Mater. Interfaces* **2017**, *9*, 1542-1552.

[20] Z. Cao, W. Wu, Y. Li, J. Zhao, W. He, J. Liu, H. Zhang, G. Li, *Ionics* **2020**, *26*, 4247-4256.

[21] S. Song, B. Chen, Y. Ruan, J. Sun, L. Yu, Y. Wang, J. Thokchom, *Electrochim. Acta* **2018**, *270*, 501-508.

[22] S. Kobi, A. Mukhopadhyay, *Scripta Mater.* **2019**, *162*, 214-218.

[23] X. Ma, Y. Xu, *ACS Appl. Mater. Interfaces* **2022**, *14*, 2939-2948.

[24] A. Sodhiya, A. K. Singh, S. Soni, S. Patel, R. Kumar, *Appl. Phys. A* **2022**, *128*, 639.

[25] Y. Lu, X. Meng, J. A. Alonso, M. T. Fernández-Díaz, C. Sun, *ACS Appl. Mater. Interfaces* **2018**, *11*, 2042-2049.
